# Supplementary material for: Supported self-management for people with type 2 diabetes: a meta-review of quantitative systematic reviews
Source: BMJ Open. 2018 Dec 14;8(12):e024262. doi: 10.1136/bmjopen-2018-024262 (PMC6303627; doi:10.1136/bmjopen-2018-024262)

Supplemental Table 1 Detailed Search Terms

Basic search strategy for all databases

General SMS terms *or* LTC specific SMS terms

AND

LTC terms

AND

Quantitative or Qualitative review filter

AND

Apply human, English, and published 1993 onwards limits

All searches in [Title/Abstract]

Detailed search terms: general SMS terms

**General self-management support terms: Medline, AMED, EMBASE, PsychINFO**				
	**Medline**	**AMED**	**EMBASE**	**PsychINFO**
#1	Exp Self care/	Exp Self care/	Exp Self care/	Exp Self care skills/
#2	Exp Communication/	Exp Education professional/	Exp Health education/	Exp Self management/
#3	Exp Professional Family Relations/	Exp Education nonprofessional/	Exp Patient education/	Exp Health behavior/
#4	Exp Telephone/	Exp Human activities/	Exp Telehealth/	Exp Self efficacy/
#5	Exp Professional Patient Relations/	Exp Self concept/	Exp Interpersonal communication/	Exp Self help techniques/

#6	Exp Health education/	Exp Self help groups/	Exp Empowerment/	Exp Coping behavior/
#7	Exp Attitude of health personnel/	Exp Telemedicine/	Exp Self concept/	Exp Behavior modification/
#8	Exp Cellular phone/	Exp Communication/	Exp Patient participation/	Exp Self monitoring/
#9	Exp Patient education as topic/	Exp Rehabilitation/		Exp Health knowledge/
#10	Exp Handheld computer/	Exp Professional patient relations/		Exp Health education/
#11	Exp Self efficacy/	Exp Professional family relations/		Exp Telemedicine/
#12	Exp Activities of Daily Living/			Exp Client education/
#13	Exp Self help devices/			
#14	Exp Community health services/			
#15	Exp Rehabilitation/			
#16	(Self ADJ2 (car* or manag* or help or administ* or monitor* or medicat*)) or self-car* or self-manag* or self-help or self-administ* or self-monitor* or self-medicat* or selfcar* or selfmanagement or selfhelp or selfadminist* or selfmonitor* or selfmedicat*			
#17	SM or SMS			
#18	Responsib* or Autonom*			
#19	Manag* or copes or coping			
#20	“Disease management”			
#21	“expert patient”			
#22	(professional or clinician) ADJ2 development			
#23	Educat* or training or skill* or knowledge			
#24	Confidence or self-efficacy			
#25	(Access* or provi*) ADJ3 (information or records or results)			
#26	Monitor* or self-monitor* or selfmonitor*			
#27	((patient or individual* or person* or client*) ADJ3 (remind* or feedback))			
#28	(Tele ADJ2 (health or medicine or care)) or tele-health or tele-medicine or tele-care or telehealth or telemedicine or telecare			
#29	“Short message service” or SMS or “mobile phone” or “text message*”			
#30	(home or environment* or living or assistive) ADJ2 (adaptation or modif* or equipment or technolog*)			
#31	“Care plan*”			
#32	“Action plan*”			
#33	Hypno* ADJ1 (self or home)			

#34	(cognitive or psychological or interpersonal or relaxation or biofeedback) ADJ3 (therap* or intervention* or program*)
#35	CBT
#36	Psychoeducation*
#37	(Peer or patient or emotional or social or psychosocial) ADJ1 (support or group)
#38	“Expert patient”
#39	Financial ADJ1 control
#40	“personal health budget*”
#41	(Financial or monetary or payment* or discount or service*) ADJ5 incentiv*

**General self-management support terms: BNI, CINAHL**		
	**BNI**	**CINAHL**
#1	Exp Self care/	Exp Self care/
#2	Exp Self medication/	Exp Self concept/
#3	Exp Patients: education/	Exp Patient education/
#4	Exp Personal care/	Exp Health education/
#5	Exp Self help groups/	Exp Attitude of Health Personnel/
#6	Exp Patients: empowerment/	Exp Telehealth/
#7	Exp Interpersonal relations/	Exp Communication skills/
#8	Exp Technology in health care/	Exp Assistive technology devices/
#9	Exp Disabilities: aids and appliances/	Exp Support groups/
#10	Exp Telemedicine/	Exp Rehabilitation/
#11	Self ADJ2 (car* or manag* or help or admistrat* or monitor* or medicat*)	Self ADJ2 car*
#12	or self-car* or self-manag* or self-help or self-adminisrat* or self-monitor* or self-medicat*	Self ADJ2 manag*
#13	SM or SMS	Self ADJ2 help
#14	Responsib* or Autonom*	Self ADJ2 administrat*
#15	Manag* or copes or coping	Self ADJ2 monitor*
#16	“Disease management”	Self ADJ2 medicat*
#17	“expert patient”	self-car*
#18	(professional or clinician) ADJ2 development	self-manag*
#19	Educat* or training or skill* or knowledge	SM
#20	Confidence or self-efficacy	SMS
#21	(Access* or provi*) ADJ3 (information or records or results)	Autonom*

#22	Monitor* or self-monitor* or selfmonitor*	Responsib*
#23	((patient or individual* or person* or client*) ADJ3 (remind* or feedback))	Manag*
#24	(Tele ADJ2 (health or medicine or care)) or tele-health or tele-medicine or tele-care or telehealth or telemedicine or telecare	copes
#25	“Short message service” or SMS or “mobile phone” or “text message*”	coping
#26	(home or environment* or living or assistive) ADJ2 (adaptation or modif* or equipment or technolog*)	“Disease management”
#27	“Care plan*”	“expert patient”
#28	“Action plan*”	Professional ADJ2 development
#29	Hypno* ADJ1 (self or home)	Clinician ADJ2 development
#30	(cognitive or psychological or interpersonal or relaxation or biofeedback) ADJ3 (therap* or intervention* or program*)	Educat*
#31	CBT	knowledge
#32	Psychoeducation*	skill*
#33	(Peer or patient or emotional or social or psychosocial) ADJ1 (support or group)	training
#34	“Expert patient”	self-efficacy
#35	Financial ADJ1 control	Confidence
#36	“personal health budget*”	Access* N3 information
#37	(Financial or monetary or payment* or discount or service*) ADJ5 incentiv*	Access* N3 records
#38		Access* N3 results
#39		Monitor*
#40		Patient N3 remind*
#41		Patient N3 feedback
#42		Individual* N3 remind
#43		Individual* N3 feedback
#44		Tele N2 health
#45		Tele N2 medicine
#46		Tele N2 care
#47		“text message*”
#48		Home N2 adaptation
#49		Home N2 modif*
#50		Assistive N2 technolog*
#51		“Care plan*”
#52		“Action plan*”
#53		Hypno* N1 self

#54		Cognitive N3 therap*
#55		Psychological N3 intervention*
#56		Relaxation N3 program*
#57		CBT
#58		Psychoeducation*
#59		Peer N3 support
#60		Patient N3 group
#61		“Expert patient”
#62		Financial N1 control
#63		“personal health budget*”
#64		Financial N5 incentiv*
#65		Monetary N5 incentiv*

**1. Diabetes Mellitus SMS terms: Medline, AMED, EMBASE, PsychINFO**				**2.**
	**Medline**	**EMBASE**		
#1	Exp Blood Glucose Self Monitoring/	Exp Diabetes education/		TS= (“Exp Blood Glucose Self Monitoring”)
#2	Exercise or training or rehabilitati*			TS= (Exercise or training or rehabilitati*)
#3	(Lifestyle or occupational) ADJ1 (intervention* or modification* or therapy)			TS= (Lifestyle or occupational) NEAR/1 (intervention* or modification* or therapy)
#4	Foot care			TS= “Foot care”
#5	(Smok* or nicotine or tobacco) ADJ3 (cessation or quit*)			TS= ((Smok* or nicotine or tobacco) NEAR/3 (cessation or quit*))
#6	Diet*			TS= Diet*

**1. Diabetes Mellitus SMS terms: BNI, CINAHL**		
	**BNI**	**CINAHL**
#1	Exp Diabetes: Health promotion/	Exp Diabetic diet/
#2		Exp Diabetic foot/
#3		Exp Diabetes Education/
#4	“Foot care”	“Foot care”
#5	(Smok* or nicotine or tobacco) ADJ3 (cessation or quit*)	Smok* N3 cessation
#6	Diet*	Diet*

LTC terms

1. Diabetes Mellitus LTC terms: Medline, AMED, EMBASE, PsychINFO				
	**Medline**	**AMED**	**EMBASE**	**PsychINFO**
#1	Diabetes mellitus, type 1/	Exp Diabetes Mellitus/	Diabetes Mellitus/	Diabetes Mellitus/
#2	Diabetes mellitus, type 2/			
#3	Insulin resistance/			
#4	Diabetic ketoacidosis/			
#5	(diabet* or dm) ADJ5 (typ* ADJ3 (one or 1 or I))			
#6	(diabet* or dm) ADJ5 (typ* ADJ3 (two or 2 or II))			
#7	(Insulin or noninsulin or non-insulin) ADJ2 (resistan* or depend*)			
#8	Diabet*			
#9	DM or DM1 or DM2 or T1D or T1DM or T2D or T2DM or NIDDM or IDDM or MODY			
#1(	"Glucose ?tolerance"			

1. Diabetes Mellitus LTC Terms: BNI, CINAHL		
	**BNI**	**CINAHL**
#1	Diabetes/	Diabetes mellitus, type 1/
#2		Diabetes mellitus, type 2/
#3		Diabetic patients/
#4	diabet* or dm	Diabet* N5 1
#5	(Insulin or noninsulin or non-insulin) ADJ2 (resistan* or depend*)	Diabet* N5 I
#6	DM1 or DM2 or T1D or T1DM or T2D or T2DM or NIDDM or IDDM or MODY	Diabet* N5 one
#7		DM N5 I
#8		Diabet* N5 2
#9		Diabet* N5 II
#10		Diabet* N5 two
#11		DM N5 II
#12		Insulin N2 resistan*
#13		Insulin N2 depend*
#14		Non-insulin N2 depend*
#15		Diabet*
#16		DM
#17		DM1
#18		DM2
#19		"Glucose ?tolerance"

Quantitative and Qualitative Review Filter

	**Quantitative and qualitative review filter: Medline, AMED, EMBASE, PsychINFO**			
	**Medline**	**AMED**	**EMBASE**	**PsychINFO**
#1	meta-analysis/	meta-analysis/	systematic review/	meta-analysis/
#2	meta analysis as topic/		meta-analysis/	literature review/
#3	review literature as topic/			
#4	MEDLINE			
#5	(systematic review* or meta-analy* or metaanaly* or "research synthesis" or “literature review”)			
#6	systematic ADJ3 literature			
#7	data ADJ2 extract*			
#8	((information or data) ADJ3 synthesis)			
#9	cochrane			
#10	(qualitative or narrative or thematic or evidence or realist or interpret* or induct* or refutational or framework or systematic or textual) adj2 (approach or review* or synthes* or meta-summary or “meta summary” or summary)			
#11	Meta adj1 (summary or narrative or synthesis or ethnograph* or study or data or interpretation or aggregation or needs-assessment or ‘needs assessment’)			
#12	meta-summary or meta-narrative or meta-synthesis or meta-ethnograph* or meta-study or meta-data-analysis or meta-data-synthesis or meta-interpretation or meta-aggregation			
#13	‘reciprocal translational analysis’			
#14	‘lines-of-arg?ment synthesis’ or ‘lines of arg?ment synthesis’			
#15	‘LOA synthesis’			
#16	‘grounded formal theory’			
#17	‘grounded theory synthesis’			
#18	ecological adj2 (triangulation or sentence or synthesis)			
#19	Phenomenography			
#20	((mixed or multi* or cross) adj1 (method* or design* or research or strategy)) adj2 (synthesis or review)			
#21	(mixed-method* or multi-method* or mixed-design or multi-design or multiple-methods or multi-strategy or cross-design) adj2 (synthesis or review)			
#22	Bayesian adj1 (meta-analysis or ‘meta analysis’)			
#23	‘case survey’			
#24	“qualitative comparative analysis”			
#25	Or/ 1-25			
#26	letter.pt.	Letter.pt	letter.pt	-

#27	comment.pt.	Comment.pt or commentary.pt	-	-
#28	editorial.pt.	editorial.pt.	editorial.pt	-
#29	Or/26-28			
#30	25 not 29			

Supplemental Table 2 Exclusion Criteria

	**Exclusion criterion**
1	Exclude if it is not written in English
2	Exclude if does not include human participants
3	Exclude reviews published before 1993
4	Exclude if it is not a systematic review of the literature
5	Exclude if the paper does not focus on, or include one or more of the exemplar LTCs.
6	Exclude if the focus is not about self-management support interventions
7	Exclude if the systematic review does not include RCTs in the search strategy
8	Exclude if does not measure one of the following outcomes: Use of healthcare services (including scheduled and unscheduled use of healthcare services and hospital admission rates), health outcomes (including biological markers of disease), symptoms, health behaviour, quality of life or self-efficacy
9	Exclude if the paper is a published conference abstract, thesis, protocol, or summary of other reviews
10	Exclude if the paper is a shorter and less detailed version of a Cochrane review or if there has been an updated version of it published
11	Exclude if unable to data extract the information on RCTs in the selected LTC separately from the rest of the findings
12	Exclude if already included in original review
13	Withdrawn

Table 3. Systematic reviews excluded at full text screening (Update)

Afable, A. and Karingula, N. S. (2016) 'Evidence based review of type 2 diabetes prevention and management in low and middle income countries', 7(10), pp. 209–229. doi: 10.4239/wjd.v7.i10.209.	6. Focus is not on self-management support interventions
Arafat, Y., Izham, M. and Ibrahim, M. (2016) 'Using the transtheoretical model to enhance self-management activities in patients with type 2 diabetes : a systematic review'. doi: 10.1111/jphs.12138	11. Unable to data extract information on RCTs for T2D separately from the rest of the findings
Asante, E. (2013) 'Interventions to promote treatment adherence in type 2 diabetes mellitus', British Journal of Community Nursing, 18(6), pp. 267–274. doi: 10.12968/bjcn.2013.18.6.267.	7. Does not include RCTs in the search strategy
Avery, L., Flynn, D., A, van W., FF, S. and MI, T. (2012) 'Changing Physical Activity Behavior in Type 2 Diabetes: A systematic review and meta-analysis of behavioral interventions.', *Diabetes Care*. 35(12), pp. 2681–2689. doi: 10.2337/dc11-2452.	6. Focus is not on self-management support interventions
Baig, A. A., Benitez, A., Quinn, M. T. and Burnet, D. L. (2016) 'Family interventions to improve diabetes outcomes for adults', 1353(1), pp. 89–112. doi: 10.1111/nyas.12844.Family.	11. Unable to data extract information on RCTs for T2D separately from the rest of the findings
Baron, J., Mcbain, H. and Newman, S. (2012) 'The Impact of Mobile Monitoring Technologies on Glycosylated Hemoglobin in Diabetes: A Systematic Review', *Journal of Diabetes Science and Technology*, 6(5), pp. 1185–1196.	6. Focus is not on self-management support interventions
Boren SA, Gunlock TL, Peeples MM, Krishna S: Computerized learning technologies for diabetes: a systematic review. Journal of Diabetes Science & Technology 2008, 2:139-146.	11. Unable to data extract information on RCTs for T2D separately from the rest of the findings
Bhurji, N., Javer, J., Gasevic, D. and Khan, N. A. (2016) 'Improving management of type 2 diabetes in South Asian patients : a systematic review of intervention studies'. doi: 10.1136/bmjopen-2015-008986.	6. Focus is not on self-management support interventions
Bonner, T., Foster, M. and Spears-Lanoix, E. (2016) 'Type 2 diabetes–related foot care knowledge and foot self-care practice interventions in the United States: a systematic review of the literature', *Diabetic Foot & Ankle*, 7(1),	6. Focus is not on self-management support interventions

p. 29758. doi: 10.3402/dfa.v7.29758.	
Brzan, P. P. (2016) ‘Mobile Applications for Control and Self Management of Diabetes : A Systematic Review’, *Journal of Medical Systems*. Journal of Medical Systems. doi: 10.1007/s10916-016-0564-8.	4. Not a systematic review of the literature
Buhi, E. R., Trudnak, T. E., Martinasek, M. P., Oberne, A. B., Fuhrmann, H. J. and Mcdermott, R. J. (no date) ‘Mobile phone-based behavioural interventions for health : A’, pp. 1–20. doi: 10.1177/0017896912452071.	6. Focus is not on self-management support interventions
Brown, S. A. (1992) ‘Meta-analysis of diabetes patient education research: Variations in intervention effects across studies’, Research in Nursing & Health, 15(6), pp. 409–419.	7. Does not include RCTs in the search strategy
Carter, B. M., Barba, B. and Kautz, D. D. (2013) ‘Culturally tailored education for African Americans with type 2 diabetes.’, Medsurg nursing, 22, pp. 105–123.	7. Does not include RCTs in the search strategy
Cassimatis, M. and Kavanagh, D. J. (2012) ‘Effects of type 2 diabetes behavioural telehealth interventions on glycaemic control and adherence: a systematic review.’, *Journal of telemedicine and telecare*, 18(8), pp. 447–50. doi: 10.1258/jtt.2012.GTH105.	11. Unable to data extract information on RCTs for T2D separately from the rest of the findings
Chen, C.-M. and Chang Yeh, M. (2015) ‘The experiences of diabetics on self-monitoring of blood glucose: a qualitative metasynthesis.’, *Journal of Clinical Nursing*. 24(5/6), pp. 614–626. doi: 10.1111/jocn.12691.	6. Focus is not on self-management support interventions
Cimo, A., Stergiopoulos, E., Cheng, C., Bonato, S. and Dewa, C. S. (2012) ‘Effective lifestyle interventions to improve type II diabetes self-management for those with schizophrenia or schizoaffective disorder : a systematic review’.	11. Unable to data extract information on RCTs for T2D separately from the rest of the findings
Cochran, J. and Conn, V. S. (2008) ‘Meta-analysis of Quality of Life Outcomes Following Diabetes Self-management Training’, The Diabetes Educator, 34(5), pp. 815–823.	7. Does not include RCTs in the search strategy
Cotter, A. P., Durant, N., Agne, A. A. and Cherrington, A. L. (2014) ‘Internet interventions to support lifestyle modification for diabetes management: A systematic review of the evidence’, Journal of Diabetes and its Complications. Elsevier Inc., 28(2), pp. 243–251. doi: 10.1016/j.jdiacomp.2013.07.003.	11. Unable to data extract information on RCTs for T2D separately from the rest of the findings

Cox, D. J., Taylor, A. G., Dunning, E. S., Winston, M. C., Luk Van, I. L., McCall, A., Singh, H. and Yancy, W. S. (2013) ‘Impact of Behavioral Interventions in the Management of Adults with Type 2 Diabetes Mellitus’, Current Diabetes Reports, 13(6), pp. 860–868. doi: 10.1007/s11892-013-0423-7.	4. Not a systematic review of the literature
Creamer, J., Attridge, M., Ramsden, M., Cannings-John, R. and Hawthorne, K. (2016) ‘Culturally appropriate health education for Type 2 diabetes in ethnic minority groups: an updated Cochrane Review of randomized controlled trials.’, Diabetic Medicine. 33(2), pp. 169–183. doi: 10.1111/dme.12865.	10. Paper is a shorter and less detailed version of a Cochrane review or if there is an updated version published
David, S. K. and Rafiullah, M. R. M. (2016) ‘Innovative health informatics as an effective modern strategy in diabetes management: a critical review’, International Journal of Clinical Practice, 70(6), pp. 434–449. doi: 10.1111/ijcp.12816.	4. Not a systematic review of the literature
Deakin, T. A., McShane, C. E., Cade, J. E. and Williams, R. (2005) ‘Group based training for self-management strategies in people with type 2 diabetes mellitus’, in Steinsbekk, A. (ed.) Cochrane Database of Systematic Reviews. Chichester, UK: John Wiley & Sons, Ltd. doi: 10.1002/14651858.CD003417.pub2.	13. Withdrawn
de Jongh, T., Gurol-Urganci, I., Vodopivec-Jamsek, V., Car, J. and Atun, R. (2012) ‘Mobile phone messaging for facilitating self-management of long-term illnesses’, Cochrane Database of Systematic Reviews.(12). doi: 10.1002/14651858.CD007459.pub2.	5. Does not focus on or include T2D
Debussche, X. (2014) ‘Is adherence a relevant issue in the self-management education of diabetes? A mixed narrative review’, Diabetes, Metabolic Syndrome and Obesity: Targets and Therapy, p. 357. doi: 10.2147/DMSO.S36369.	7. Does not include RCTs in the search strategy
Dennis, S. M., Harris, M., Lloyd, J., Powell Davies, G., Faruqi, N. and Zwar, N. (2013) ‘Do people with existing chronic conditions benefit from telephone coaching? A rapid review.’, Australian Health Review. 37(3), pp. 381–388. doi: 10.1071/AH13005.	11. Unable to data extract information on RCTs for T2D separately from the rest of the findings
Dombrowski, E., Fitzpatrick, A., Hall-Alston, J., Barnes, C. and Singleton, J. (2013) ‘The effect of nutrition and exercise in addition to hypoglycemic medications on HbA1C in patients with type 2 diabetes mellitus: a systematic review protocol’, JBI Database of Systematic Reviews and Implementation Reports, 11(7), pp. 400–413. doi: 10.11124/jbisrir-2013-954.	9. Published conference abstract, thesis, protocol or summary of other reviews

Dube, L., Van den Broucke, S., Housiaux, M. and Rendall-Mkosi, K. (2014) ‘Diabetes self-management education programmes in high-and low-mortality developing countries’, Journal of Endocrinology, Metabolism and Diabetes of South Africa, 19(1), p. 32.	10. Paper is a shorter and less detailed version of a Cochrane review or if there is an updated version published
Dube, L., Van den Broucke, S., Housiaux, M., Dhoore, W. and Rendall-Mkosi, K. (2015) ‘Type 2 Diabetes Self-management Education Programs in High and Low Mortality Developing Countries: A Systematic Review.’, Diabetes Educator. 41(1), pp. 69–85. doi: 10.1177/0145721714558305.	11. Unable to data extract information on RCTs for T2D separately from the rest of the findings
El-Gayar, O., Timsina, P., Nawar, N. and Eid, W. (2013) ‘A systematic review of IT for diabetes self-management: are we there yet?’, International Journal of Medical Informatics. 82(8), pp. 637–652. doi: 10.1016/j.ijmedinf.2013.05.006.	11. Unable to data extract information on RCTs for T2D separately from the rest of the findings
Elissen, A. M. J., Steuten, L. M. G., Lemmens, L. C., Drewes, H. W., Lemmens, K. M. M., Meeuwissen, J. A. C., Baan, C. A. and Vrijhoef, H. J. M. (2013) ‘Meta-analysis of the effectiveness of chronic care management for diabetes: investigating heterogeneity in outcomes.’, Journal of Evaluation in Clinical Practice. 19(5), pp. 753–762. doi: 10.1111/j.1365-2753.2012.01817.x.	11. Unable to data extract information on RCTs for T2D separately from the rest of the findings
Ellis, S. E., Speroff, T., Dittus, R. S., Brown, A., Pichert, J. W. and Elasy, T. A. (2004) ‘Diabetes patient education: a meta-analysis and meta-regression’, Patient Education and Counseling, 52(1), pp. 97–105.	11. Unable to data extract information on RCTs for T2D separately from the rest of the findings
Everson-Hock, E. S., Johnson, M., Jones, R., Woods, H. B., Goyder, E., Payne, N. and Chilcott, J. (2013) ‘Community-based dietary and physical activity interventions in low socioeconomic groups in the UK: A mixed methods systematic review.’, Preventive Medicine. 56(5), pp. 265–272. doi: 10.1016/j.ypmed.2013.02.023.	6. Focus is not on self-management support interventions
Figueira, F., Umpierre, D., Cureau, F., Zucatti, A., Dalzochio, M., Leitão, C. and Schaan, B. (2014) ‘Association between Physical Activity Advice Only or Structured Exercise Training with Blood Pressure Levels in Patients with Type 2 Diabetes: A Systematic Review and Meta-Analysis.’, Sports Medicine. 44(11), pp. 1557–1572. doi: 10.1007/s40279-014-0226-2.	6. Focus is not on self-management support interventions

Fitzner, K. K., Heckinger, E., Tulas, K. M., Specker, J. and McKoy, J. (2014) ‘Telehealth Technologies: Changing the Way We Deliver Efficacious and Cost-Effective Diabetes Self-Management Education.’, Journal of Health Care for the Poor & Underserved. 25(4), pp. 1853–1897. doi: 10.1353/hpu.2014.0157.	11. Unable to data extract information on RCTs for T2D separately from the rest of the findings
Fitzpatrick, S. L., Schumann, K. P. and Hill-Briggs, F. (2013) ‘Problem solving interventions for diabetes self-management and control: A systematic review of the literature.’, Diabetes Research & Clinical Practice. 100(2), pp. 145–161. doi: 10.1016/j.diabres.2012.12.016.	11. Unable to data extract information on RCTs for T2D separately from the rest of the findings
Flodgren, G., Rachas, A., Farmer, A. J., Inzitari, M. and Shepperd, S. (2015) ‘Interactive telemedicine: effects on professional practice and health care outcomes’, Cochrane Database of Systematic Reviews. (9). doi: 10.1002/14651858.CD002098.pub2.	6. Focus is not on self-management support interventions
Franz, M. J., Boucher, J. L., Rutten-Ramos, S. and VanWormer, J. J. (2015) ‘Lifestyle Weight-Loss Intervention Outcomes in Overweight and Obese Adults with Type 2 Diabetes: A Systematic Review and Meta-Analysis of Randomized Clinical Trials.’, Journal of the Academy of Nutrition & Dietetics. 115(9), pp. 1447–1463. doi: 10.1016/j.jand.2015.02.031.	6. Focus is not on self-management support interventions
Fuchs, S., Henschke, C., Blüme, M. and Busse, R. (2014) ‘Disease Management Programs for Type 2 Diabetes in Germany.’, Deutsches Aerzteblatt International. 111(26), pp. 453–463. doi: 10.3238/arztebl.2014.0453.	7. Does not include RCTs in the search strategy
Garabedian, L., Ross-Degnan, D., Wharam, J., Garabedian, L. F. and Wharam, J. F. (2015) ‘Mobile Phone and Smartphone Technologies for Diabetes Care and Self-Management.’, Current Diabetes Reports. 15(12), pp. 1–9. doi: 10.1007/s11892-015-0680-8.	4. Not a systematic review of the literature
Glazier, R. H., Bajcar, J., Kennie, N. R. and Willson, K. (2006) ‘A Systematic Review of Interventions to Improve Diabetes Care in Socially Disadvantaged Populations’, Diabetes Care, 29(7), pp. 1675–1688.	11. Unable to data extract information on RCTs for T2D separately from the rest of the findings
Gonzalez, L. S., Berry, D. C. and Davison, J. A. (2013) ‘Diabetes Self-Management Education Interventions and Glycemic Control Among Hispanics: A Literature Review.’, Hispanic Health Care International. 11(4), pp. 157–166. doi: 10.1891/1540-4153.11.4.157.	11. Unable to data extract information on RCTs for T2D separately from the rest of the findings

Gucciardi, E., Chan, V. W.-S., Manuel, L. and Sidani, S. (2013) 'A systematic literature review of diabetes self-management education features to improve diabetes education in women of Black African/Caribbean and Hispanic/Latin American ethnicity.', Patient Education & Counseling. 92(2), pp. 235–245. doi: 10.1016/j.pec.2013.03.007.	11. Unable to data extract information on RCTs for T2D separately from the rest of the findings
Hall, A. K., Cole-Lewis, H. and Bernhardt, J. M. (2015) 'Mobile text messaging for health: a systematic review of reviews.', Annual Review of Public Health. 36(1), pp. 393–415. doi: 10.1146/annurev-publhealth-031914-122855.	4. Not a systematic review of the literature
Hamine, S., Gerth-Guyette, E., Faulx, D., Green, B. B. and Ginsburg, A. S. (2015) 'Impact of mHealth Chronic Disease Management on Treatment Adherence and Patient Outcomes: A Systematic Review.' Journal of Medical Internet Research. 17(2), pp. e52–e52. doi: 10.2196/jmir.3951.	6. Focus is not on self-management support interventions
Hayashino, Y., JL, J., Fukumori, N., Nakamura, F. and Fukuhara, S. (2012) 'Effects of supervised exercise on lipid profiles and blood pressure control in people with type 2 diabetes mellitus: A meta-analysis of randomized controlled trials.', Diabetes Research & Clinical Practice. 98(3), pp. 349–360. doi: 10.1016/j.diabres.2012.10.004.	6. Focus is not on self-management support interventions
Hill-Briggs F, Gemmell L: Problem solving in diabetes self-management and control: a systematic review of the literature. Diabetes Educator 2007, 33:1032-1050;	11. Unable to data extract information on RCTs for T2D separately from the rest of the findings
Hisashige, A. (2012) 'The Effectiveness and Efficiency of Disease Management Programs for Patients with Chronic Diseases', Global Journal of Health Science, 5(2), pp. 27–48. doi: 10.5539/gjhs.v5n2p27.	4. Not a systematic review of the literature
Hoerth, R. R. and Udlis, K. (2014) 'Effectiveness of Group Education for Persons with Type 2 Diabetes Mellitus : A Systematic Review of the Literature', *AADE in Practice*, pp. 28–38.	11. Unable to data extract information on RCTs for T2D separately from the rest of the findings
Holtz, B. and Lauckner, C. (2012) 'Diabetes Management via Mobile Phones: A Systematic Review', Telemedicine and e-Health, 18(3), pp. 175–184. doi: 10.1089/tmj.2011.0119.	6. Focus is not on self-management support interventions

Hou, Y., Lin, L., Li, W., Qiu, J., Zhang, Y. and Wang, X. (2015) 'Effect of combined training versus aerobic training alone on glucose control and risk factors for complications in type 2 diabetic patients: a meta-analysis.', International Journal of Diabetes in Developing Countries. 35(4), pp. 524–532. doi: 10.1007/s13410-015-0329-9.	6. Focus is not on self-management support interventions
Housden, L., Wong, S. T. and Dawes, M. (2013) 'Effectiveness of group medical visits for improving diabetes care: a systematic review and meta-analysis', Canadian Medical Association Journal, 185(13), pp. E635–E644. doi: 10.1503/cmaj.130053.	6. Focus is not on self-management support interventions
Huang, Z., Tao, H., Meng, Q. and Jing, L. (2015) 'MANAGEMENT OF ENDOCRINE DISEASE: Effects of telecare intervention on glycemic control in type 2 diabetes: a systematic review and meta-analysis of randomized controlled trials', European Journal of Endocrinology, 172(3), pp. R93–R101. doi: 10.1530/EJE-14-0441.	6. Focus is not on self-management support interventions
Huang, X., Pan, J., Chen, D., Chen, J., Chen, F. and Hu, T. (2016) 'Efficacy of lifestyle interventions in patients with type 2 diabetes: A systematic review and meta-analysis', European Journal of Internal Medicine. European Federation of Internal Medicine., 27, pp. 37–47. doi: 10.1016/j.ejim.2015.11.016.	6. Focus is not on self-management support interventions
Hunt, C. W. (2015) 'Technology and diabetes self-management: An integrative review', World Journal of Diabetes, 6(2), p. 225. doi: 10.4239/wjd.v6.i2.225.	4. Not a systematic review of the literature
Jalil, S., Myers, T. and Atkinson, I. (2015) 'A Meta-Synthesis of Behavioral Outcomes from Telemedicine Clinical Trials for Type 2 Diabetes and the Clinical User-Experience Evaluation (CUE)', Journal of Medical Systems, 39(3), p. 28. doi: 10.1007/s10916-015-0191-9.	6. Focus is not on self-management support interventions
Jones, A., Gladstone, B. P., Lübeck, M., Lindekilde, N., Upton, D. and Vach, W. (2014) 'Motivational interventions in the management of HbA1c levels: A systematic review and meta-analysis', Primary Care Diabetes. 8(2), pp. 91–100. doi: 10.1016/j.pcd.2014.01.009.	11. Unable to data extract information on RCTs for T2D separately from the rest of the findings
Joo, J. Y. (2014) 'Effectiveness of Culturally Tailored Diabetes Interventions for Asian Immigrants to the United States. A Systematic Review', Diabetes Educator. College of Nursing. 40(5), pp. 605–615. doi: 10.1177/0145721714534994.	6. Focus is not on self-management support interventions

Kim, S. H. and Lee, A. (2016) 'Health-Literacy-Sensitive Diabetes Self-Management Interventions: A Systematic Review and Meta-Analysis.', Worldviews on Evidence-Based Nursing. 13(4), pp. 324–333. doi: 10.1111/wvn.12157.	11. Unable to data extract information on RCTs for T2D separately from the rest of the findings
Klein, H. A., Jackson, S. M., Street, K., Whitacre, J. C. and Klein, G. (2013) 'Diabetes Self-Management Education: Miles to Go', Nursing Research and Practice, 2013, pp. 1–15. doi: 10.1155/2013/581012.	4. Not a systematic review of the literature
Krishna S, Boren SA: Diabetes self-management care via cell phone: a systematic review. Journal of Diabetes Science & Technology 2008, 2:509-517.	7. Does not include RCTs in the search strategy
Lau, Y., Htun, T. P., Wong, S. N., Wilson Tam, W. S., Klainin-Yobas, P. and Tam, W. S. W. (2016) 'Efficacy of Internet-Based Self-Monitoring Interventions on Maternal and Neonatal Outcomes in Perinatal Diabetic Women: A Systematic Review and Meta-Analysis.', Journal of Medical Internet Research. 18(8), pp. e220–e234. doi: 10.2196/jmir.6153.	11. Unable to data extract information on RCTs for T2D separately from the rest of the findings
Lepard, M. G., Joseph, A. L., Agne, A. A. and Cherrington, A. L. (2015) 'Diabetes self-management interventions for adults with type 2 diabetes living in rural areas: a systematic literature review.', Current Diabetes Reports. 15(6), p. 608. doi: 10.1007/s11892-015-0608-3.	11. Unable to data extract information on RCTs for T2D separately from the rest of the findings
Li, S. X., Ye, Z., Whelan, K. and Truby, H. (2016) 'The effect of communicating the genetic risk of cardiometabolic disorders on motivation and actual engagement in preventative lifestyle modification and clinical outcome: a systematic review and meta-analysis of randomised controlled trials', British Journal of Nutrition, 116(05), pp. 924–934. doi: 10.1017/S0007114516002488.	6. Focus is not on self-management support interventions
Loveman E, Frampton GK, Clegg AJ: The clinical effectiveness of diabetes education models for Type 2 diabetes: a systematic review. Health Technology Assessment (Winchester, England) 2008, 12:1-116, iii.	11. Unable to data extract information on RCTs for T2D separately from the rest of the findings

Machado Menezes, M., Takáo Lopes, C. and de Souza Nogueira, L. (2016) 'Impact of educational interventions in reducing diabetic complications: a systematic review.', Revista Brasileira de Enfermagem. 69(4), pp. 726–737. doi: 10.1590/0034-7167.2016690422i.	11. Unable to data extract information on RCTs for T2D separately from the rest of the findings
Maez, L., Erickson, L. and Naumuk, L. (2014) 'Diabetic education in rural areas.', Rural & Remote Health. University of Cincinnati, Cincinnati, Ohio, USA: James Cook University, Rural Health Research Unit, 14(2), pp. 1–7.	6. Focus is not on self-management support interventions
March, S., Torres, E., Ramos, M., Ripoll, J., García, A., Bulilete, O., Medina, D., Vidal, C., Cabeza, E., Llull, M., Zabaleta-del-Olmo, E., Aranda, J. M., Sastre, S. and Llobera, J. (2015) 'Adult community health-promoting interventions in primary health care: A systematic review', Preventive Medicine. Elsevier Inc., 76, pp. S94–S104. doi: 10.1016/j.ypmed.2015.01.016.	11. Unable to data extract information on RCTs for T2D separately from the rest of the findings
Marcolino, M. S., Maia, J. X., Alkmim, M. B. M., Boersma, E. and Ribeiro, A. L. (2013) 'Telemedicine Application in the Care of Diabetes Patients: Systematic Review and Meta-Analysis', PLoS ONE. 8(11), p. e79246. doi: 10.1371/journal.pone.0079246.	11. Unable to data extract information on RCTs for T2D separately from the rest of the findings
Martínez-González, N. A., Tandjung, R., Djalali, S. and Rosemann, T. (2015) 'The impact of physician-nurse task shifting in primary care on the course of disease: a systematic review.', Human Resources for Health. BioMed Central, 13(1), p. 55. doi: 10.1186/s12960-015-0049-8.	4. Not a systematic review of the literature
McDermott, M. S. and While, A. E. (2013) 'Maximizing the healthcare environment: A systematic review exploring the potential of computer technology to promote self-management of chronic illness in healthcare settings', Patient Education and Counseling, 92(1), pp. 13–22. doi: 10.1016/j.pec.2013.02.014.	11. Unable to data extract information on RCTs for T2D separately from the rest of the findings
Mignerat, M., Lapointe, L. and Vedel, I. (2014) 'Using telecare for diabetic patients: A mixed systematic review', Health Policy and Technology. 3(2), pp. 90–112. doi: 10.1016/j.hlpt.2014.01.004.	6. Focus is not on self-management support interventions
Mulimba, A. A. C. and Byron-Daniel, J. (2014) 'Motivational interviewing-based interventions and diabetes mellitus.', British Journal of Nursing. 23(1), pp. 8–14.	11. Unable to data extract information on RCTs for T2D separately from the rest of the findings

Mushcab, H., Kernohan, W. G., Wallace, J. and Martin, S. (2015) 'Web-Based Remote Monitoring Systems for Self-Managing Type 2 Diabetes: A Systematic Review.', Diabetes Technology & Therapeutics. 17(7), pp. 498–509. doi: 10.1089/dia.2014.0296.	6. Focus is not on self-management support interventions
Nam, S., PhD, R. N., Janson, S., DNSc, R. N., Stotts, N., EdD, R. N., Chesla, C., DNSc, R. N., Kroon, L. and PharmD, C. D. E. (2012) 'Effect of Culturally Tailored Diabetes Education in Ethnic Minorities With Type 2 Diabetes: A Meta-analysis.', J Cardiovasc Nurs. 27(6), pp. 505–518. doi: 10.1097/JCN.0b013e31822375a5.	12. Already in original review
Navarro-Flores, E., Gijón-Noguerón, G., Cervera-Marín, J. A. and Labajos-Manzanares, M. T. (2015) 'Assessment of Foot Self-Care in Patients With Diabetes', Foot & Ankle Specialist, 8(5), pp. 406–412. doi: 10.1177/1938640015585963.	11. Unable to data extract information on RCTs for T2D separately from the rest of the findings
Noordman, J., van der Weijden, T. and van Dulmen, S. (2012) 'Communication-related behavior change techniques used in face-to-face lifestyle interventions in primary care: A systematic review of the literature', Patient Education and Counseling. 89(2), pp. 227–244. doi: 10.1016/j.pec.2012.07.006.	11. Unable to data extract information on RCTs for T2D separately from the rest of the findings
Nordheim, L. V., Haavind, M. T. and Iversen, M. M. (2014) 'Effect of telemedicine follow-up care of leg and foot ulcers: a systematic review.', BMC Health Services Research. BioMed Central, 14(1), p. 565. doi: 10.1186/s12913-014-0565-6.	6. Focus is not on self-management support interventions
Norris SL, Zhang X, Avenell A, Gregg E, Bowman B, Serdula M, Brown TJ, Schmid CH, Lau J: Long-term effectiveness of lifestyle and behavioral weight loss interventions in adults with type 2 diabetes: a meta-analysis. American Journal of Medicine 2004, 117:762-774.	6. Focus is not on self-management support interventions
Or, C. K. L. and Tao, D. (2014) 'Does the use of consumer health information technology improve outcomes in the patient self-management of diabetes? A meta-analysis and narrative review of randomized controlled trials.', International Journal of Medical Informatics. 83(5), pp. 320–329. doi: 10.1016/j.ijmedinf.2014.01.009.	11. Unable to data extract information on RCTs for T2D separately from the rest of the findings
Pal, K., Eastwood, S. V, Michie, S., Farmer, A., Barnard, M. L., Peacock, R., Wood, B., Edwards, P. and Murray, E. (2014) 'Computer-based interventions to improve self-management in adults with type 2 diabetes: a systematic review and meta-analysis.', *Diabetes Care*. 37(6), pp. 1759–1766. doi: 10.2337/dc13-1386.	10. Paper is a shorter and less detailed version of a Cochrane review or if there is an updated version published

Panagioti, M., Richardson, G., Small, N., Murray, E., Rogers, A., Kennedy, A., Newman, S. and Bower, P. (2014) ‘Self-management support interventions to reduce health care utilisation without compromising outcomes: a systematic review and meta-analysis’, BMC Health Services Research, 14(1), p. 356. doi: 10.1186/1472-6963-14-356.	11. Unable to data extract information on RCTs for T2D separately from the rest of the findings
Pansier, B. and Schulz, P. J. (2015) ‘School-based diabetes interventions and their outcomes: a systematic literature review’, Journal of Public Health Research, 4(1). doi: 10.4081/jphr.2015.467.	5. Does not focus on or include T2D
Pennington, M., Visram, S., Donaldson, C., White, M., Lhussier, M., Deane, K., Forster, N. and Carr, S. M. (2013) ‘Cost-effectiveness of health-related lifestyle advice delivered by peer or lay advisors : synthesis of evidence from a systematic review’, pp. 1–12. doi: 10.1136/bmj.f2618.	6. Focus is not on self-management support interventions
Pereira, K., Phillips, B., Johnson, C. and Vorderstrasse, A. (2015) ‘Internet delivered diabetes self-management education: a review.’, Diabetes Technology & Therapeutics. New Rochelle, New York: Mary Ann Liebert, Inc., 17(1), pp. 55–63. doi: 10.1089/dia.2014.0155.	11. Unable to data extract information on RCTs for T2D separately from the rest of the findings
Pimouguet C, Le Goff M, Thiebaut R, Dartigues JF, Helmer C: Effectiveness of disease-management programs for improving diabetes care: a meta-analysis. CMAJ Canadian Medical Association Journal 2011, 183:E115-127.	
Plotnikoff, R. C., Costigan, S. A., Karunamuni, N. D. and Lubans, D. R. (2013) ‘Community-Based Physical Activity Interventions for Treatment of Type 2 Diabetes: A Systematic Review with Meta-Analysis’, Frontiers in Endocrinology, 4(January), pp. 1–17. doi: 10.3389/fendo.2013.00003.	6. Focus is not on self-management support interventions
Posadzki, P., Lee, M. S. and Ernst, E. (2012) ‘Complementary and alternative medicine for diabetes mellitus: an overview of systematic reviews.’, Focus on Alternative & Complementary Therapies. 17(3), pp. 142–148. doi: 10.1111/j.2042-7166.2012.01159.x.	4. Not a systematic review of the literature
Quiñones, A. R., Richardson, J., Freeman, M., Fu, R., O’Neil, M. E., Motu’apuaka, M. and Kansagara, D. (2014) ‘Educational group visits for the management of chronic health conditions: a systematic review.’, Patient Education & Counseling. 95(1), pp. 3–29. doi: 10.1016/j.pec.2013.12.021.	11. Unable to data extract information on RCTs for T2D separately from the rest of the findings

Radhakrishnan, K. (2012) 'The efficacy of tailored interventions for self-management outcomes of type 2 diabetes, hypertension or heart disease: a systematic review.', Journal of Advanced Nursing. 68(3), pp. 496–510. doi: 10.1111/j.1365-2648.2011.05860.x.	6. Focus is not on self-management support interventions
Riazi, H., Larijani, B., Langarizadeh, M. and Shahmoradi, L. (2015) 'Managing diabetes mellitus using information technology: a systematic review', Journal of Diabetes & Metabolic Disorders. Journal of Diabetes & Metabolic Disorders, 14(1), p. 49. doi: 10.1186/s40200-015-0174-x.	11. Unable to data extract information on RCTs for T2D separately from the rest of the findings
Ricci-Cabello, I., Ruiz-Perez, I., Nevot-Cordero, A., Rodriguez-Barranco, M., Sordo, L. and Goncalves, D. C. (2013) 'Health Care Interventions to Improve the Quality of Diabetes Care in African Americans: A systematic review and meta-analysis', Diabetes Care, 36(3), pp. 760–768. doi: 10.2337/dc12-1057.	11. Unable to data extract information on RCTs for T2D separately from the rest of the findings
Ricci-Cabello, I., Ruiz-Perez, I., Rojas-García, A., Pastor, G. and Gonçalves, D. C. (2013) 'Improving Diabetes Care in Rural Areas: A Systematic Review and Meta-Analysis of Quality Improvement Interventions in OECD Countries', PLoS ONE. 8(12), p. e84464. doi: 10.1371/journal.pone.0084464.	11. Unable to data extract information on RCTs for T2D separately from the rest of the findings
Rice, K., Te Hiwi, B., Zwarenstein, M., Lavallee, B., Barre, D. E. and Harris, S. B. (2016) 'Best Practices for the Prevention and Management of Diabetes and Obesity-Related Chronic Disease among Indigenous Peoples in Canada: A Review', Canadian Journal of Diabetes. Elsevier Inc., 40(3), pp. 216–225. doi: 10.1016/j.jcjd.2015.10.007.	11. Unable to data extract information on RCTs for T2D separately from the rest of the findings
Sanders, A. R. J., van Weeghel, I., Vogelaar, M., Verheul, W., Pieters, R. H. M., de Wit, N. J. and Bensing, J. M. (2013) 'Effects of improved patient participation in primary care on health-related outcomes: a systematic review', Family Practice, 30(4), pp. 365–378. doi: 10.1093/fampra/cmt014.	6. Focus is not on self-management support interventions
Sarkisian CA, Brown AF, Norris KC, Wintz RL, Mangione CM: A systematic review of diabetes self-care interventions for older, African American, or Latino adults. Diabetes Educator 2003, 29:467-479.	11. Unable to data extract information on RCTs for T2D separately from the rest of the findings
Sazlina, S., Browning, C. and Yasin, S. (2013) 'Interventions to Promote Physical Activity in Older People with Type 2 Diabetes Mellitus: A Systematic Review', Frontiers in Public Health, 1(December), pp. 1–13. doi: 10.3389/fpubh.2013.00071	6. Focus is not on self-management support interventions

Schellenberg, E. S., Dryden, D. M., Vandermeer, B., Ha, C. and Korownyk, C. (2013) 'Lifestyle Interventions for Patients With and at Risk for Type 2 Diabetes: A Systematic Review and Meta-analysis.', Annals of Internal Medicine. Philadelphia, Pennsylvania: American College of Physicians, 159(8), pp. 543–551. doi: 10.7326/0003-4819-159-8-201310150-00007.	6. Focus is not on self-management support interventions
Sharoni, S. K. A, Minhat, H. S., Mohd Zulkefli, N. A. and Baharom, A. (2016) 'Health education programmes to improve foot self-care practices and foot problems among older people with diabetes: a systematic review', International Journal of Older People Nursing, 11(3), pp. 214–239. doi: 10.1111/opn.12112.	7. Does not include RCTs in the search strategy
Sherifali, D., Bai, J.-W., Kenny, M., Warren, R. and Ali, M. U. (2015) 'Diabetes self-management programmes in older adults: a systematic review and meta-analysis.', Diabetic Medicine. 32(11), pp. 1404–1414. doi: 10.1111/dme.12780.	11. Unable to data extract information on RCTs for T2D separately from the rest of the findings
Sinclair, C. (2015) 'Effectiveness and User Acceptance of Online Chronic Disease Management Interventions in Rural and Remote Settings: Systematic Review and Narrative Synthesis', Clinical Medicine Insights: Therapeutics, 7, p. CMT.S18553. doi: 10.4137/CMT.S18553	7. Does not include RCTs in the search strategy
Smalls, B. L., Walker, R. J., Bonilha, H. S., Campbell, J. A. and Egede, L. E. (2015) 'Community Interventions to Improve Glycemic Control in African Americans with Type 2 Diabetes: A Systemic Review', Global Journal of Health Science, 7(5), pp. 171–182. doi: 10.5539/gjhs.v7n5p171.	7. Does not include RCTs in the search strategy
Steinsbekk, A., Rygg, L. Ø., Lisulo, M., Rise, M. B. and Fretheim, A. (2012) 'Group based diabetes self-management education compared to routine treatment for people with type 2 diabetes mellitus. A systematic review with meta-analysis.', BMC health services research, 12(1), p. 213. doi: 10.1186/1472-6963-12-213.	12. Already in original review
Stellefson, M., Chaney, B., Barry, A. E., Chavarria, E., Tennant, B., Walsh-Childers, K., Sriram, P. S. and Zagora, J. (2013) 'Web 2.0 chronic disease self-management for older adults: a systematic review.', Journal of Medical Internet Research. 15(2), pp. e35–e35. doi: 10.2196/jmir.2439.	11. Unable to data extract information on RCTs for T2D separately from the rest of the findings
Stellefson, M., Dipnarine, K. and Stopka, C. (2013) 'The chronic care model and diabetes management in US primary care settings: a systematic review.', Preventing Chronic Disease. 10, pp. E26–E26. doi: 10.5888/pcd10.120180.	11. Unable to data extract information on RCTs for T2D separately from the rest of the findings

Sturt, J., Dennick, K., Hessler, D., Hunter, B. M., Oliver, J. and Fisher, L. (2015) 'Effective interventions for reducing diabetes distress: systematic review and meta-analysis.', International Diabetes Nursing. 12(2), pp. 40–55. doi: 10.1179/2057332415Y.0000000004.	11. Unable to data extract information on RCTs for T2D separately from the rest of the findings
Su, D., McBride, C., Zhou, J. and Kelley, M. S. (2016) 'Does nutritional counseling in telemedicine improve treatment outcomes for diabetes? A systematic review and meta-analysis of results from 92 studies.', Journal of Telemedicine & Telecare. pp. 333–347. doi: 10.1177/1357633X15608297.	6. Focus is not on self-management support interventions
Su, D., Zhou, J., Kelley, M. S., Michaud, T. L., Siahpush, M., Kim, J., Wilson, F., Stimpson, J. P. and Pagán, J. A. (2016) 'Does telemedicine improve treatment outcomes for diabetes? A meta-analysis of results from 55 randomized controlled trials.116, pp. 136–148. doi: 10.1016/j.diabres.2016.04.019.	6. Focus is not on self-management support interventions
Suksomboon, N., Poolsup, N. and Nge, Y. L. (2014) 'Impact of Phone Call Intervention on Glycemic Control in Diabetes Patients: A Systematic Review and Meta-Analysis of Randomized, Controlled Trials', PLoS ONE. 9(2), p. e89207. doi: 10.1371/journal.pone.0089207.	6. Focus is not on self-management support interventions
Sultana, F., Srilekha, S. and Soumendra, S. (2015) 'Cost effectiveness of exercise intervention and lifestyle counselling in prevention and control of diabetes mellitus-a review', International Journal of Pharma and Bio Sciences, 6(4), pp. B566–B576.	6. Focus is not on self-management support interventions
Sumlin, L. L. and Garcia, A. A. (2012) 'Effects of Food-Related Interventions for African American Women with Type 2 Diabetes', The Diabetes Educator, 38(2), pp. 236–249. doi: 10.1177/0145721711422412.	6. Focus is not on self-management support interventions
Tan, C. C. L., Cheng, K. K. F. and Wang, W. (2015) 'Self-care management programme for older adults with diabetes: An integrative literature review', International Journal of Nursing Practice, 21, pp. 115–124. doi: 10.1111/ijn.12388.	11. Unable to data extract information on RCTs for T2D separately from the rest of the findings
Terranova, C. O., Brakenridge, C. L., Lawler, S. P., Eakin, E. G. and Reeves, M. M. (2015) 'Effectiveness of lifestyle-based weight loss interventions for adults with type 2 diabetes: a systematic review and meta-analysis', Diabetes, Obesity and Metabolism, 17(4), pp. 371–378. doi: 10.1111/dom.12430.	6. Focus is not on self-management support interventions

Thongsai, S. and Youjaiyen, M. (2013) ‘The Long-Term Impact of Education on Diabetes for Older People: A Systematic Review’, Global Journal of Health Science, 5(6), pp. 30–39. doi: 10.5539/gjhs.v5n6p30.	11. Unable to data extract information on RCTs for T2D separately from the rest of the findings
Thorpe, C. T., Fahey, L. E., Johnson, H., Deshpande, M., Thorpe, J. M. and Fisher, E. B. (2013) ‘Facilitating Healthy Coping in Patients with Diabetes: A Systematic Review.’, Diabetes Educator. 39(1), pp. 33–52. doi: 10.1177/0145721712464400.	8. Does not measure one of the following outcomes: Health outcomes, symptoms, health behaviour, quality of life or self-efficacy
Timm, M., Rodrigues, M. C. S. and Machado, V. B. (2013) ‘Adherence to treatment of type 2 diabetes mellitus: a systematic review of randomized clinical essays’, Journal of Nursing UFPE on line, 7(4), pp. 1204–1215. doi: 10.5205/1981.	6. Focus is not on self-management support interventions
Toma, T., Athanasiou, T., Harling, L., Darzi, A. and Ashrafian, H. (2014) ‘Online social networking services in the management of patients with diabetes mellitus: systematic review and meta-analysis of randomised controlled trials.’, Diabetes Research & Clinical Practice. Elsevier Science, 106(2), pp. 200–211. doi: 10.1016/j.diabres.2014.06.008.	6. Focus is not on self-management support interventions
Tricco, A. C., Ivers, N. M., Grimshaw, J. M., Moher, D., Turner, L., Galipeau, J., Halperin, I., Vachon, B., Ramsay, T., Manns, B., Tonelli, M. and Shojania, K. (2012) ‘Effectiveness of quality improvement strategies on the management of diabetes: a systematic review and meta-analysis’, The Lancet. 379(9833), pp. 2252–2261. doi: 10.1016/S0140-6736(12)60480-2.	6. Focus is not on self-management support interventions
Tshiananga, J. K. T., Kocher, S., Weber, C., Erny-Albrecht, K., Berndt, K. and Neeser, K. (2012) ‘The Effect of Nurse-led Diabetes Self-management Education on Glycosylated Hemoglobin and Cardiovascular Risk Factors: A Meta-analysis.’, Diabetes Educator. 38(1), pp. 108–123. doi: 10.1177/0145721711423978.	11. Unable to data extract information on RCTs for T2D separately from the rest of the findings
Uchendu, C. and Blake, H. (2017) ‘Effectiveness of cognitive-behavioural therapy on glycaemic control and psychological outcomes in adults with diabetes mellitus: a systematic review and meta-analysis of randomized controlled trials’, Diabetic Medicine, 34(3), pp. 328–339. doi: 10.1111/dme.13195.	6. Focus is not on self-management support interventions

Vaes, A. W., Cheung, A., Atakhorrami, M., Groenen, M. T. J., Amft, O., Franssen, F. M. E., Wouters, E. F. M. and Spruit, M. A. (2013) ‘Effect of “activity monitor-based” counseling on physical activity and health-related outcomes in patients with chronic diseases: A systematic review and meta-analysis’, Annals of Medicine, 45(5–6), pp. 397–412. doi: 10.3109/07853890.2013.810891.	6. Focus is not on self-management support interventions
Van Huffel, L., Tomson, C. R. V, Ruige, J., Nistor, I., Van Biesen, W. and Bolignano, D. (2014) ‘Dietary Restriction and Exercise for Diabetic Patients with Chronic Kidney Disease: A Systematic Review’, PLoS ONE. 9(11), p. e113667. doi: 10.1371/journal.pone.0113667.	6. Focus is not on self-management support interventions
Vasconcelos, H. C. A. de, Freitas, R. W. J. F. de, Marinho, N. B. P., Damasceno, M. M. C., Araújo, T. L. de and Lima, F. E. T. (2013) ‘Effectiveness of telephone interventions as a strategy for glycemic control: an integrative literature review’, Texto & Contexto - Enfermagem, 22(1), pp. 239–246. doi: 10.1590/S0104-07072013000100029.	6. Focus is not on self-management support interventions
Vernooij, R. W. M., Willson, M. and Gagliardi, A. R. (2016) ‘Characterizing patient-oriented tools that could be packaged with guidelines to promote self-management and guideline adoption: a meta-review.’, Implementation Science. 11, pp. 1–13. doi: 10.1186/s13012-016-0419-1.	4. Not a systematic review of the literature
Walker, R. J., Smalls, B. L., Bonilha, H. S., Campbell, J. A. and Egede, L. E. (2013) ‘Behavioral interventions to improve glycemic control in African Americans with type 2 diabetes: a systematic review’, Ethnicity and Disease, 23(4), pp. 401–408.	11. Unable to data extract information on RCTs for T2D separately from the rest of the findings
Werfalli, M., Raubenheimer, P., Engel, M., Peer, N., Kalula, S., Kengne, A. P. and Levitt, N. S. (2015) ‘Effectiveness of community-based peer-led diabetes self-management programmes (COMP-DSMP) for improving clinical outcomes and quality of life of adults with diabetes in primary care settings in low and middle-income countries (LMIC): a systematic review and meta-analysis’, BMJ Open, 5(7), p. e007635. doi: 10.1136/bmjopen-2015-007635.	9. Published conference abstract, thesis, protocol or summary of other reviews
Whittemore R: Culturally competent interventions for Hispanic adults with type 2 diabetes: a systematic review. Journal of Transcultural Nursing 2007, 18:157-166.	7. Does not include RCTs in the search strategy

Wildevuur, S. E. and Simonse, L. W. (2015) ‘Information and Communication Technology–Enabled Person-Centered Care for the “Big Five” Chronic Conditions: Scoping Review’, Journal of Medical Internet Research, 17(3), p. e77. doi: 10.2196/jmir.3687.	4. Not a systematic review of the literature
Worswick, J., Wayne, S. C., Bennett, R., Fiander, M., Mayhew, A., Weir, M. C., Sullivan, K. J. and Grimshaw, J. M. (2013) ‘Improving quality of care for persons with diabetes: an overview of systematic reviews - what does the evidence tell us?’, Systematic Reviews, 2(1), p. 26. doi: 10.1186/2046-4053-2-26.	4. Not a systematic review of the literature
Zeh, P., Sandhu, H. K., Cannaby, A. M. and Sturt, J. A. (2012) ‘The impact of culturally competent diabetes care interventions for improving diabetes-related outcomes in ethnic minority groups: a systematic review’, Diabetic Medicine, 29(10), pp. 1237–1252. doi: 10.1111/j.1464-5491.2012.03701.x.	11. Unable to data extract information on RCTs for T2D separately from the rest of the findings
Zhai, Y.-K., Zhu, W.-J., Cai, Y.-L., Sun, D.-X. and Zhao, J. (2014) ‘Clinical- and cost-effectiveness of telemedicine in type 2 diabetes mellitus: a systematic review and meta-analysis.’, Medicine. 93(28), pp. e312–e312. doi: 10.1097/MD.0000000000000312.	6. Focus is not on self-management support interventions
Zimbudzi, E., Lo, C., Misso, M., Ranasinha, S. and Zoungas, S. (2015) ‘Effectiveness of management models for facilitating self-management and patient outcomes in adults with diabetes and chronic kidney disease’, Systematic Reviews. Systematic Reviews, 4(1), p. 81. doi: 10.1186/s13643-015-0072-9.	4. Not a systematic review of the literature

Supplemental Table 4 Summary table of characteristics of included studies and main findings

Review	Intervention of Interest	Participant demographics	Setting and delivery mode	Content, focus and mode of instruction	Duration, intensity and follow-up	Comparison	Main Results	Main conclusion and important quality concerns
**Bolen 2014 [35]**<br>138 RCTs n=33,124<br>Search dates to Nov 2011<br>R-AMSTAR 39	Patient activation interventions (PAIs behavioural interventions to engage patients in care) on type 2 diabetes complications and glycaemic control	WM age 59y (112 RCTs), WM BMI 33kg/m2 (89 RCTs), baseline HbA1c (8.1%/65mmol/mol) SysBP 140mmHg, Duration DM mean 10y<br>RCTs from US (48%); Europe (32%): 25 in UK. Also: 14 countries globally	Primary care 31%, DM clinic 11%, Home (in person, online, phone) 19% Not reported 26%.<br>Delivery: team of physicians (48%), nurses (44%), dieticians (28%), educators (17%).	PAIs: problem solving, audit and feedback, individualised care plans, financial incentive, peer support/family, lay health advisor/community health worker, psychological counselling, theory-based counselling, skill building	Median intended sessions 9; Median contact time: 1.5h/session.<br><br>Mean study FU: 12m (range: 3-96m).	Usual care/ minimal intervention<br><br>Usual care in RCTs not summarised	**Meta-analysis**<br>HbA1c: WMD -0.37% (4mmol/mol) [95% CI -0.28 to -0.45] SysBP: WMD -2.2mmHg 95% CI [-1.0 to -3.5] Weight: WMD -2.3 lbs [95% CI -1.3 to -3.2] LDL: WMD -4.2 mg/dL [95% CI -1.5, 6.9]<br>No intervention strategy outperformed any other in adjusted meta-regression.	PAIs modestly decreased HbA1c.<br>Most RCTs judged moderate or high quality.<br>No one intervention strategy had a significantly larger impact on HbA1c.<br>*Publication bias in HbA1c outcomes, however, no change in point estimate or CI after sensitivity analysis*
**Chodosh 2005 [13]**<br>26 RCTs n=2579<br>Search dates 1983-2004<br>R-AMSTAR 34	LTC SM: interventions to improve active participation in self-monitoring and/or decision-making	NR	NR	NR	Only studies with FU 3-12m included	Usual care, conventional diet advice, diabetes pamphlet or consultation	**Meta-analysis**<br>Compared with control, significant reduction in: HbA1c (ES -0.36) and blood glucose (ES -0.28) but not weight	LTC SM programmes improved glycaemic control. Feedback associated with improved HbA1c<br><br>*Possible publication bias.*
**Chrvala 2016 [41]**<br>120 RCTs n=2,2947<br>Search dates 1997–2013<br>R-AMSTAR 31	SM interventions to reduce HbA1c.	Intervention: n=11,854 mean age: 58.5y mean HbA1c: 8.55% (70mmol/mol)<br>Control: n=11,093 mean age: 58.7y mean HbA1c 8.48% (69mmol/mol)	Delivery by one (60%) or teams of: physicians, nurses educators, CHWs, dietitians, physical therapists, SW, pharmacists, psychologists, etc.	Individual education 41.5%, group education 29.7%, individual and group education 17.8%, remote delivery (online or telephone) 10.2%	Median duration: 6m (range 1–36). Mean contact time: 18h (range 1–460) in 92 interventions.<br><br>Median FU: 12m. (range 6w-96m).	Usual care or minimal education interventions	**Narrative**: 62% of RCTs reported significant change in HbA1c. Mean HbA1c reduction: 0.74% (8mmol/mol) (I), 0.17% (2mmol/mol) (c). Absolute reduction: 0.57% (6mmol/mol). Greater HbA1c reductions were associated with: group + individual interventions, contact ≥10h, persistently elevated HbA1c (> 9%/75mmol/mol)	SM education significantly decreased HbA1c. Mode of delivery, hours of engagement, and baseline HbA1c affect the likelihood of improving HbA1c.<br><br>*Publication bias not assessed.*

**Dale 2012 [42]**<br>10 RCTs<br>n=3,763<br>Search dates 1966 -2011<br>R-AMSTAR 32	Peer support in adults living with diabetes.	African-Americans (3 RCTs), Spanish-speaking (1 RCT). American Indians/Alaska natives (1 RCT) Ethnicity NR (8 RCTs).<br>RCTs from: USA: 10, UK: 3, Ireland: 1	Settings: NR<br>Delivered by: nurses, physicians, diabetes educators, dieticians, physical therapists, SW, CHW, pharmacists, psychologists.	Education, lifestyle, social/ emotional support, goal setting, behaviour change, problem solving, communication<br>Mode: group (8 RCTs), group + peer phone calls (2 RCTs), peer phone calls (1 RCT), online peer interaction (2 RCTs)	Variable duration and intensity: telecare for 150d-1y 12w web programme; community groups over 6w to 2y<br>FU: median 6m range: 2-24m	Usual care<br><br>Usual care in RCTs not summarised	**Narrative:** Compared to controls, significant improvements in:<br>HbA1c (3 of 14 RCTs); BP (1 of 4 RCTs); cholesterol (1 of 6 RCTs); BMI (2 of 7 RCTs), Self-efficacy (2 of 3RCTs)<br>No consistent pattern of effect related to any model of peer support.	Peer support benefited some adults with type 2 diabetes Quality scores for the majority of studies were 'fair to good'.<br><br>*Publication bias not assessed.*
**Duke 2009 [53]**<br>9 RCTs<br>n=1359<br>Search dates 1996-2007<br>R-AMSTAR 36	Individual patient education systematic programmes delivered face to face	Men and women in all but 1 RCT. Mean age 52-65y. One study focussed on a low literacy migrant population	Delivery mostly by diabetes educators and dieticians. One RCT trained a lay link worker.	Education, diet and exercise, medication compliance, glucose self-monitoring, diabetes complications, foot care, services available, motivation and behaviour strategies.	Most RCTs involved 2-4h face-to-face time. 2 RCTs <2h contact and 2 RCTs >5h of contact.	Usual care or group education.	**Meta-analysis**: Group education more effective than individual education (WMD HbA1c 0.8%/ 9mmol/mol)). No difference in BP or BMI outcomes.<br><br>Individual education may be most effective if HbA1c >8% (9mmol/mol). Impact on QoL unclear.	Group education more effective than individual in reducing HbA1c short term. Individual education may be more effective for people with higher baseline HbA1c.<br><br>RCTs generally poor quality with majority having high risk of bias.<br><br>*Publication bias not assessed.*
**Ekong 2016 [44]**<br>14 RCTs<br>n=4066<br>Search dates to Oct 2014<br>R-AMSTAR: 31	MI as a behaviour change intervention.	Demographic summary NR but included new diagnoses, uncontrolled diabetes and obese patients with diabetes.<br><br>RCTs from: Thailand, Netherlands, USA, Taiwan, Denmark, UK	Setting: primary care, doctors' offices, community health facilities<br><br>Delivery: GPs, nurses, dieticians, psychologists, diabetes educators.	MI tailored to patient preference/behaviour target, MI counselling only, MI added to education or usual care. Diet (7 RCTs), activity (6 RCTs). Smoking /drinking (4 RCTs).<br><br>Face to face (11 RCTs), group (1 RCT), FU phone calls (3 RCTS)	Median duration: 12m (range 3-24m) Contact time: 30-90m, frequency: 1 to 5 times<br><br>FU NR	Usual care or non-MI interventio n e.g. health education or behavioural weight program	**Narrative**: Significant improvements in HbA1c (4 of 14 RCTs), BP (1of 6 RCTs), dietary behaviour (5 of 7 RCTs), BMI (1 of 8 RCTs), self-management behaviour (1 of 3 RCTs) No significant differences for physical activity, smoking cessation, alcohol reduction, cholesterol or waist circumference.	Improvements observed in some clinical and behavioural outcomes.<br><br>High heterogeneity of included RCTs makes it difficult to conclude that MI should be implemented.<br><br>Comparison of methods, outcomes and maintaining fidelity are difficult.<br><br>*Publication bias not assessed.*
**Fan 2009 [47]**<br>50 RCTs<br>1990-2006<br>R-AMSTAR 20	Which intervention elements, mode and delivery are most	Adults >18 with T2D.<br><br>RCTs from Australia, Canada,	Setting: outpatient clinics, community clinics, home, hospital	18% of RCTs focused on 1 T2D self-management topic<br>82% covered >1 topic	Several sessions with mean of 10 (range 1-28) for mean 17 contact hours (range 1-	NR	**Meta-analysis:**<br>Compared to other intervention types:<br>*Behavioural interventions*: larger effect sizes on	Interventions may improve knowledge, self-care behaviours and metabolic control. Overall weighted mean effect size: +0.56. Effect size

	effective for improving knowledge, self-management behaviour and metabolic control	Finland, Germany, Italy, Korea, Netherlands, Taiwan, UK, US		Group (40%), one-to-one 32%, mixed (28%)	52) over mean 22 weeks (range 1-48). 32% RCTs delivered a booster session.		metabolic (ES 0.63) and self-care outcomes (ES 0.92). *Psychological interventions*: moderate effects on self-care behaviour (ES=0.67) and metabolic control (ES=0.40). *Mixed interventions*: larger effect for knowledge (ES=1.32), moderate effect for metabolic control (ES 0.50). *Educational interventions:* moderate effect on knowledge (ES 0.59)<br><br>*Mixed teaching*: larger effects for knowledge (ES 1.69) and metabolic control (ES 0.69)<br><br>*Interactive teaching:* (ES 0.54)<br><br>*HCP Interactions* (phone or face-to-face) produced larger effect sizes	greatest for knowledge gain followed by metabolic control and self-management behaviours. Interventions with more sessions and a longer duration yield larger effects for knowledge and metabolic control (not self-management behaviours). Booster sessions enhanced effectiveness of interventions<br><br>*Publication bias not assessed*
**Gary 2003 [15]** 18 RCTs n=2720 Search dates: 1984-1997 R-AMSTAR 36	Clear behavioural or counselling component aimed at improving long-term diabetes self-care behaviour.	Mean age: 57y	Setting: 96% OPD<br><br>Delivery: nurse: 39%, dietician: 26%, physician: 17%, psychologist: 9%, health educator: 4%	Diet: 70%, exercise: 57%, foot-care: 35%, medication adherence/change: 33%, SMBG and education.<br><br>Mostly group and/or individual counselling. Also phone outreach, clinician prompting, computer program and AV materials	Duration: 1-19m (median 5m). Median visits 9 (range 2-52)	Usual care or minimal interventio n	**Meta-analysis**: Strong evidence of HbA1c reduction compared with control (ES -0.43). HbA1c reduction (WMD -0.52%)(6mmol/mol). No effect: other glycaemic control measures or wt.<br><br>Physician led interventions may cause larger HbA1c than nurses or dieticians.	Educational or behavioural interventions improved glycaemic control. Physician led interventions may cause greater improvements, this may be due to manipulation of medical regimens.<br><br>*Possible publication bias*
**Heinrich 2010 [16]** 14 RCTs n=1778 Search dates: 2001-2009	Multicompone nt SM interventions targeting ≥2 behaviours or focussing on	3 RCTs specific targeted African-American and/or Latino/Hispanic adults.	Delivery: PhD student, also patients' usual HCP	Learning only: 4 RCTs, learning and planning: 7 RCTs, learning, planning and practising: 3 RCTs.	Low intensity: Usual care + pre-intervention visit+ computer lifestyle assessment). High	Usual care	**Narrative:** Diet most responsive to change regardless of intervention form. Interventions successfully increasing activity focussed on SM	Dietary change and SMBG appear reactive to multicomponent interventions. Interventions aiming to increase activity should focus on SM behaviours and lifestyle

R-AMSTAR 24	diabetes in general.	3 studies only included women.	All but 1 RCT additional to usual care.	SM: 6 RCTs; SM behaviours: 5 RCTs, lifestyle change: 3 RCTs	intensity: 2.5d retreat + 6m of weekly 4h meetings		behaviours and lifestyle change	change.<br><br>*Publication bias not assessed*
**Jonkman 2016 [46]**<br>13 RCTs<br>n=3829<br>Search dates: 1985- 2013<br>R-AMSTAR 31	Quantification of SM components on HRQoL	Mean age: 60y, female: 54%<br><br>RCTs from: USA: 9 RCTs, UK: 3 RCTs, Japan, Iceland	Delivery NR but peer interaction in 7 RCTs	Goal setting: 93%, lifestyle education: 86%, problem solving, support allocation also used<br><br>Face to face contact: 57%	Median 6 contacts (range: 1 to 35) Mean duration 5.3m, median 3m (range 1d to 24m)	Usual Care	**Meta-regression:** SM had positive effects on HRQoL at 6 and 12m. SMD 0.11 (95% CI 0.01, 0.22) SMD 0.08 (95% CI 0.02, 0.18) respectively<br>Negatively association of peer interaction with HRQoL at 6m FU (SMD 0.25, 95% Cl 0.48, 0.02).	SM interventions improve HRQoL at 6 and 12m. Effects beyond 12m need to be established.<br>Teaching problem-solving skills were positively associated with HRQoL.<br><br>*Possible publication bias*
**Minet 2010 [14]**<br>43 RCTs<br>n=7677<br>Search dates: 1988-2007<br>R-AMSTAR 37	Self-care management interventions using educational or behavioural strategies.	Mean age in behavioural psychosocial RCTs: 60.7y, 59.3y in educational technique RCTs	NR	Education: didactic (knowledge/skills acquisition)<br><br>Behavioural/psychosocial interventions: (cognitive/ behavioural/motivational approaches, psychological counselling (relaxation, problem solving or MI)	NR	No educational / behavioural intervention	**Meta-analysis:** HbA1c reduction compared with control (MD 0.36%/4mmol/mol)<br><br>HbA1c improvement greater at shorter FU.<br><br>Educational techniques more effective than behavioural/ psychosocial techniques for improving HbA1c.	Self-care management interventions improve HbA1c, suggestion of reduced long-term impact.<br><br>*Statistical analysis did not indicate publication bias*
**Newman 2004 [20]**<br>21 RCTs<br>n=2032<br>Search dates: 1997-2002<br>R-AMSTAR 23	Interventions that aim to increase patients' involvement and control in their lives with chronic illness.	NR	Most interventions led by HCPs	Various behavioural changes including both lifestyle and cognitive components	Duration of interventions varied. Maximum 58h.	Standard care or minimal intervention: e.g basic information behavioural weight control.	**Narrative**: Majority of interventions reduced HbA1c at some point, evidence that reductions can be sustained after 6m.<br><br>SM behaviours improved, little QoL effect, no difference in wellbeing	Interventions improve HbA1c and SM behaviours. Little effect on QoL, and no difference in psychological well-being<br>Long term effectiveness unclear.<br><br>*Publication bias not assessed.*
**Norris 2001[21]**<br>72 RCTs<br>n= NR<br>Search dates: 1981-1999<br>R-AMSTAR 27	Educational or multicomponent interventions where effects of educational component could be	Not summarised but RCTs heterogeneous with respect to patient population	Settings not summarised but included, home, clinic, remote.<br><br>Delivery not summarised but included dieticians,	Not summarised but educational interventions heterogeneous and multicomponent in some cases. Variety of provider types and educational media (written, oral,	Not summarised but variety of durations and intensity included.	Usual care in some RCTs-usual care constituted group education,	**Narrative**: Improved short-term glycaemic control (Vs usual care). Group support meetings may be beneficial. Beneficial effect: wt, diet. Mixed effect: QoL, BP, FC, PhA, chol	Interventions improve glycaemic control short term. Also benefits for weight loss and SM behaviours.<br><br>*Publication bias not assessed.*

	examined separately.		CHWs, peers, nursing students.	video, computer). Individual and group education included.		or dietician education.	Greater effect from collaborative, repetitive, interactive, individualised interventions. Effect of computers/ videos unclear	
**Norris 2002 [22]**<br>31 RCTs<br>n=4263<br>Search dates: 1981-1999<br>R-AMSTAR 31	Teaching individuals to manage diabetes through SM education.	Average age 55y (range 35-67y). Average baseline HbA1c 9.4% (range 6.1-12.9%) 79mmol/mol (range 43mmol/mol-117mmol/mol)	Mostly clinic, also home/senior centre.<br><br>Delivery: physician+ team 25%; team: 20%; nurse 13% dietician 13%; self (e.g. computer instruction) 7%, lay HCW 3%; NR 20%.	Main focus: lifestyle and knowledge. Skills (SMBG and foot care) uncommon.<br><br>Mode of instruction: collaborative 87%; theory based 39%, individual 32%; primary care 13%, computer instruction 6%	Median duration 6m (range 1-27m)<br><br>Median contacts 6 (range 1-36)<br>Median contact time 9.2h (range 1-28h)	Usual Care/less intensive interventio n e.g. individual dietician sessions	**Meta-analysis.** Improved GHb after 4m+ compared with control (ES -0.26%)<br>On average, 23.6h contact between educator and patient needed for 1% (11mmol/mol) reduction in GHb. Contact time only significant predictor of effect.	SM of education interventions improve glycaemic control short term.<br>No study fulfilled all reviewer quality criteria for bias.<br><br>*Publication bias not assessed.*
**Patil 2016 [38]**<br>17 RCTs<br>n=4715<br>Search dates: 1960 to 2015<br>R-AMSTAR 34	Effect of peer support on glycaemic control.	Population: African-American, ethnic majorities, Hispanic, White.<br><br>RCTs from Argentina, Canada, China, Europe, US.	Setting NR<br>Delivered by peer supporter	Most studies include lifestyle counselling, goal setting and behavioural and social support as peer support interventions.	Group sessions: every 1m-3m, regular phone calls from peers. Face to face sessions ranged from as needed to every 3m.	Usual care and minimal interventio n control considered separately in sub-analyses	**Meta-analysis**: Overall HbA1c improved by 0.24%(95% CI: 0.05-0.43%) (2mmol/mol). SMD of 0.12 (95% CI 0.03-0.22) (1mmol/mol) (intervention Vs control) Hispanic population: HbA1c -0.48% (95% CI, 0.25%-0.70%) (-5mmol/mol)<br>Minority participants: -0.53% (95% CI, 0.32%-0.73%). (-6mmol/mol)<br>Intervention Vs usual care control: SMD 0.15 (95% CI .02-0.27) Intervention Vs minimal intervention: SMD 0.04 (95%CI -0.07-0.15)	Peer support achieved a statistically significant but minor improvement in HbA1c. These interventions may be particularly effective in improving glycaemic control in minority groups, especially those of Hispanic ethnicity.
**Pillay 2015 [48]**	Behavioural programs for people with T2D	Australia, Canada, Germany, Hong Kong, Japan, Korea, Netherlands, UK, US	Mixture of community engagement (50 RCTs) and none(82 RCTs)<br><br>Delivery: Mixture of individual only, group only and mixed	Behavioural programs were defined as multicomponent T2D specific program with repeated interactions w trained individuals over 4 weeks<br><br>Self-management interventions had to	Most studies had FU of ≤6m,12m (8 RCTs)<br><br>Studies showing clinically important effects had:	Usual care	Behavioural interventions Vs usual care:<br>End of intervention:<br>MD -0.35 (95% CI -0.56, -0.14) 8715 ppts<br><br>6m post intervention:<br>-0.16 (95% CI -0.36, 0.04) 4138 ppts	T2D self-management education offering ≤10hrs contact time provided little benefit. Behavioural programs benefit people with sub-optimal or poor glycaemic control more than good control.<br><br>Most lifestyle and T2D self-management education and

			Mixture of face-to face, technology and mixed<br><br>Deliver personnel either 1. Non-HCP, 2. On HCP 3. MDT of HCPs	include at least one structured dietary or physical activity intervention with another component.	Mean total duration of 8m. (range 2-12m) Mean contact hours (26.4 hrs (range 7-40.5hrs) ≤10 hrs (2 RCTs)11-26 contact hrs (6 RCTs), ≥26 contact hrs (6 RCTs).		12m post intervention -0.14(95% CI -0.4, 0.12) 1494 ppts<br><br>Behavioural interventions Vs active controls<br><br>End of intervention: -0.24% (95% CI -0.41, -0.07)7518 ppts<br><br>6m post intervention -0.19% (95% CI -0.37, -0.01)595 ppts<br><br>12m post intervention -1.1% (95% CI -2.56, 0.36) 486 ppts<br><br>Behavioural changes in BMI VS usual care Post-intervention: -0.51 kg/m2 (95% CI -0.66, -0.36) 4280 ppts<br><br>6m post intervention -0.21kg/m2 (95% CI -0.32, -0.01) 1840 ppts<br>12m post intervention -0.92kg/m2 (95% CI -1.44, -0.04) 867 ppts.	support programs (usually offering >11 hrs) led to clinically important improvements in HbA1c (≥0.4% reduction).<br><br>Most T2D self-management programs without added support (especially if ≤10 contact hours) provide little benefit.<br><br>Programs with higher effect sizes were more often delivered in person than via technology.<br><br>Greater glycaemic reduction in ppts with HbA1c ≥7%, adults <65 yrs and minority groups.<br><br>Lifestyle programs led to biggest reduction in BMI.
**Qi 2015 [28]** 13 RCTs n=2352 Search dates: 1978-2014 R-AMSTAR 35	Peer support as an adjunct to existing resources to encourage self-management.	63.2% women, mean age 57.4y (range 45.7 – 67.7y) Mean HbA1c 8.2% (range 6.7-10.1%)<br><br>Studies from US (11 RCTs), Ireland (1 RCT) Vietnam (1 RCT)	Community health services (10 RCTs), public health clinics (1 RCT), Diabetes OPD (1 RCT), church (1 RCT)<br><br>Delivery: peer led group structured education (9 RCTs), one to one peer support FU.	ADA recommendations: diabetes basics, SMBG, complications, diet, exercise, medication (9 RCTs). One to one goal setting as FU (2 RCTs). Individual peer support for SM skills, social/emotional support, lifestyle change, medication (4 RCTs)	Median duration 6m (Range 3-24m)<br><br>Frequency: High: 6 RCTs (>2 contacts/ m/pt), Moderate: 2 RCTs (1-2 contacts/m/pt), Low: 5 RCTs (<1 contacts/m/pt)	Usual care, Enhanced Usual care	**Meta-analysis**: MD HbA1c −0.57 95% CI−0.78, −0.3.<br><br>High frequency contact: MD HbA1c −0.75% (95% CI −1.21, −0.29) (-8mmol/mol); moderate: −0.52% (95% CI −0.60, −0.44) -6mmol/mol; low: −0.32% (95% CI −0.74, 0.09) (-3mmol/mol). Greater reduction if baseline HbA1c ≥8.5%(≥69mmol/mol)	Peer support had a significant impact on HbA1c. Moderate/ high frequency peer support targeting poor control (HbA1c >7.5%/58mmol/mol) may be more effective than low frequency programmes for overall population. Individual intervention might be more effective than structured group intervention + individual on-going support.

							−0.78% (95% CI−1.06, −0.51) -8mmol/mol. Individual: −0.91%(95% CI −1.10, −0.71). (-10mmol/mol) Group education: −0.42% (95% CI−0.72, −0.11) (-4mmol/mol), group + individual: −0.52 (95% CI−0.66, −0.38) (6mmol/mol) ($p < 0.05$ subgroup difference).<br><br>No difference for peer support mode, location, duration.	*Possible publication bias noted*
**Sherifali 2016 [31]**<br>8 RCTS<br>n=724<br>Search dates: 1946- 2015<br>R-AMSTAR 33	Effects of health coaching on clinical outcome in adults with type 2 diabetes	Mean age: 53-66y(I) I. Women: 13%-100% (I), 36%-100% I. Mean type 2 diabetes duration: 2.7-13.1y (I)I. RCTs: Australia, Finland, S. Korea, Turkey, USA	Setting NR<br><br>Delivery: health coach, remote internet/DVD coaching mediated by nurse, PA.	Self-care knowledge, goal setting with coach. DVDs, booklets, phone coaching, remote patient reporting lifestyle SM program, face-to-face coaching	Median duration: 6.5m (range 3-16 m)<br><br>Median FU:6m (range 3-16m)	Traditional education	**Meta-analysis** Overall pooled effect of health coaching was statistically significant HbA1c reduction: -0.32% (95% CI, −0.50, −0.15). Coaching >6 m: - 0.57% (95% CI, −0.76, −0.38),(-6mmol/mol) Vs ≤6m (−0.23% 95% CI, −0.37, −0.09) (-2mmol/mol)	type 2 diabetes health coaching improved glycaemic control. Greatest effects seen for durations >6m. Coaching may be of greater benefit when offered in addition to existing care.<br>*Publication bias not assessed.*
**Sigurdardottir 2007 [51]**<br>18 RCTs<br>n=4293<br>Search dates: 2001-2005<br>R-AMSTAR 26	Education that aims to enhance diabetes-related self-care.	NR	Delivery: nurses, physicians, team of health-care providers and dieticians	Collaborative teaching methods (goal setting, problem solving, cognitive reframing). Diabetes knowledge, self-care skills (diet, exercise, drugs, psychosocial/emotional aspects). Face to face (17 RCTs) Group + individual education (13 RCTs) used most commonly.	Twelve interventions used more than 11 hours of intervention, Duration of interventions: 8 weeks – 12 months.	Usual care/ minimal intervention e.g. compressed version of intervention	**Meta-analysis** Strong evidence of reduction in HbA1c compared with control.<br>There is strong evidence to suggest greater reduction in HbA1c in individuals with baseline HbA1c ≤ 8% (≤64mmol/mol)	Educational interventions improve glycaemic control. Greater reduction in those with high baseline HbA1c.<br>*Publication bias not assessed.*

**Song 2014 [32]**<br>10 RCTs<br>n=2947<br>Search dates to Jan 2014<br>R-AMSTAR 28	The effect of MI on type 2 diabetes SM	Patients with severe medical conditions or complications excluded.	Setting: NR<br><br>Delivery: trained nurses (9 RCTs) and psychologist (1 RCT)	Intervention formulated by interviewer then: 1. Pts assisted in strengthening internal motivation for behaviour change. 2. Pts assisted in consolidating their commitment and behaviour change.	Median 4.5sessions (range 3-8). Phone + face to face -2 RCTs (14-16 contacts)<br><br>Median duration:6m (range 6-18m) FU ("time of assessment: median: 0m (range 0-3m)	Traditional type 2 diabetes health education. E.g. collective class	**Meta-Analysis** Subgroup analysis showed short-term MI (6m) significantly decreased HbA1c but no advantage for long-term MI. SM ability (diet, exercise, medication adherence, SMBG, foot care, hypo/hyperglycaemic prevention /management) significantly better in MI group than control (WMD – 2.37% (95% CI, 1.77, 2.98) p < 0.00001). -26mmol/mol	MI associated with improved SM abilities. Short-term MI (6m) effectively decreased HbA1c.<br>no mention of FU*. Unclear if "period" was duration and "time of assessment" included FU. Not clear if sub analyses made distinction between duration and duration+FU.<br><br>*Publication bias not assessed.*
**Steinsbekk 2012 [52]**<br>21RCTs<br>n=2833<br>Search dates: 1988-2007<br>R-AMSTAR 37	Group-based diabetes education	40% male.<br>Baseline average age=60y (SD 9.5), BMI 31.5kg/m2 (SD 5.6), diabetes duration was 8.1y (SD 7.0y), HbA1c 8.23% (66mmol/mol) (SD 1.80%), 81.9% used insulin and/or oral hypoglycaemic agents.	PC (12 RCTs), hospital (5 RCTs), NR (4 RCTs). HCP educators (except 2 RCTs (lay health advisors/CHWs))<br><br>Delivery: physicians +HCP (dietician, nurse, CHW, specialist nurse) Solo dietician, nurse, nutritionist less frequent.	Family member or friend invited to attend the programme (4 RCTs)	Range: 3h/y for 2y to 6-20h group-based education over 4w-10m (10 RCTs). Most intensive programme = 96h in 6m.	Routine treatment, enhanced routine treatment (individual GP/dieticia n/ nutritionist sessions.	**Meta-analysis** Very strong evidence of HbA1c effect short term (SMD -0.44%/-4mmol/mol), 12m (SMD -0.4%/-4mmol/mol), long term (SMD -0.87%/-9mmol/mol). Some evidence of self-efficacy benefit (SMD 0.28%3mmol/mol) and SM behaviour (SMD 0.55/6mmol/mol). Suggestive evidence of long term weight benefit (SMD 1.66kg). No evidence for QoL, BP, cholesterol or mortality.<br><br>Combining different educators, baseline HbA1c ≥7%/≥53mmol/mol; inviting family/friends=reduced effect	Group-based education improves glycaemic control short and long term. Some evidence of benefit on self-efficacy, SM behaviours and weight.<br>2RCTs low risk of bias, 12 RCTs moderate risk of bias and 7 RCTs high risk of bias.<br><br>*Publication bias not assessed.*
**Van Dam 2005 [54]**<br>6 RCTs<br>n=712<br>Search dates: 1991-2002<br>R-AMSTAR 31	Social support interventions (emotional, appraisal, informational or tangible assistance)	Mean age=59.3y (range 52.4 – 68y)	Delivery: peer counsellor, personal coach. Physician	Peer-patient support in group visits to physician, peer group support: phone calls from peer counsellor, internet peer group with personal coach, support from spouse/family/friends in	Frequency: weekly or monthly.<br>Duration: 5 sessions to 2y intervention period.	Usual care or education without social support interventio n.	**Narrative**: No beneficial effect of social support on glycaemic control<br><br>Social support to helps increase SM behaviour, lifestyle adjustments and psychosocial functioning. Spouse support may help	Social support does not improve glycaemic control, but may increase SM behaviours, weight loss and psychosocial well-being.<br><br>*Publication bias not assessed.*

				type 2 diabetes education.			weight loss in women only.	
**Zhang 2016 [36]**<br>20 RCTs<br>n=4494<br>Search dates to Nov 2014<br>R-AMSTAR 33	Effects of peer support on glycaemic control. Effects of different providers, types of support and intervention duration.	57.5% female. Diabetes type was unspecified in 4 RCTS but included as mean age >30y. Mexican-American (3 RCTs). African American (2 RCTs), Hispanic/Spanish speaking or Latino participants (3 RCTs)	Home, community settings, phone.<br><br>Delivery: 1. Peer leaders: -12 RCTs. 2. CHWs w similar background to pts -6 RCTs. 3 Peer-partners: pts helping/sharing experience together in groups without a leader -2 RCTs	Peer support: home -3 RCTs, curriculum -2 RCTs, curriculum-combined-reinforcement combination (regular interventions: phone calls, postcards, face-to-face, group meetings, home visits)-7 RCTs, Phone dominant -4 RCTs. Groups for goal setting, community education -4 RCTs	Duration:1.25 to 24m<br><br>5 RCTs >1FU interval (9-12m). 11 RCTs had no FU	Usual care or minimal interventio n e.g. dietician appt or 4w structured education course	**Meta-analysis** HbA_{1c} pooled effect 0.16%, 95% CI 0.25 to 0.007% (2mmol/mol)<br><br>HbA_{1c} (during intervention) 0.37% (95% CI 0.59 to 0.15). 4mmol/mol. Immediate post intervention FU: 0.21% (95% CI 0.31 to 0.11) 2mmol/mol ($P < 0.001$). 1-6m FU NS. >6m FU showed opposite result. NS difference between 4 groups.	Peer support appears effective in improving glycaemic control but effect weakens over time.<br><br>*No evidence of publication bias found by authors.*
**Medically-specific interventions**								
**Dorresteijn 2014 [43]**<br>RCTS 12<br>n=3167<br>Search dates: 1986-2010<br>R-AMSTAR 39	Educational programmes that aim to promote foot care (FC) and to prevent occurrence of foot lesions.	All patients had either T1D or type 2 diabetes<br><br>4/12 RCTs: high risk of foot ulceration, low/medium ulceration risk (4 RCTs), risk NR (4 RCTs)<br><br>RCTs in Australia, Brazil UK, USA	Community: 4RCTs. Primary care (DM OPD, hospital OPD, academic OPD) 3RCTs, secondary care (4 RCTs), ED (1 RCT)	Education including FC (3 RCTs); tailored FC education (2 RCTS), intensive FC program (6 RCTs) FC video (2 RCTs), equipment provision (2 RCTs), phone reminder (1 RCT), podiatry FU (1RCT).<br><br>Group/one to one education, FC handouts, phone reminders, hands on sessions.	10m session to 14h group education. Some interventions were single sessions + hand-outs/ FU visits/ weekly reminder phone calls.<br><br>Median FU=6m, (range 1m-7y)	Usual care, risk assessment alone, less proactive intervention	**Narrative:** Evidence is lacking that education alone can improve incidence of diabetic foot complications. Suggestion that FC knowledge and self-care behaviour improved short term.	FC education alone not effective. Future interventions should be more intensive, tailored and integrated with other interventions.<br><br>High or unclear risk of bias in all but 1 RCT. Studies underpowered with too many methodological flaws to make conclusions including clear evidence of no effect.<br><br>*Possible publication bias noted.*

**Li 2011 [18]**<br>Two RCTs<br>n=207<br>Search dates: 2002-2005<br>R-AMSTAR 41	Educations programmes (or programmes which include education) for people with DKD	Average age:63.0±13.5y (I) and 60.9±11.7y I. Overall more men than women. Diabetes duration: 20.5± 13.0y (I) 22.0± 11.7y I. T1D or type 2 diabetes all pts stage V of CKD on dialysis >30m.	Dialysis units, HD or PD unit (1RCT); OPD (1 RCT)<br><br>Delivery: specialist nurses, dieticians (1 RCT); diabetes care manager (1RCT)	SM and self-monitoring, motivational coaching (1 RCT), general discussion about living with diabetes (1 RCT)<br><br>Group-based programme (1 RCT); NR (1 RCT)	Duration: 12m SM education 3 times/w (HD units) monthly (PD units). Motivational coaching every 1-2w (HD), monthly (PD).	Usual care	**Narrative:** Interventions may improve some aspects of QoL/SM behaviours<br><br>Unclear effect on self-efficacy<br><br>No effect on mortality found.	Educational programmes for people with DKD may improve some aspects of QoL and SM behaviours.<br><br>*Insufficient studies identified to examine publication bias*
**Mcbain 2016 [26]**<br>1 RCT<br>n=64<br>Search dates to March 2016<br>R-AMSTAR 39	SM interventions specifically tailored for people with type 2 diabetes and severe mental illness.	Schizophrenia (n=46), schizoaffective disorder (n=9). Mean age (onset mental illness): 28y, type 2 diabetes mean duration: 9y. 68% oral drugs, 12% diet, 7% insulin, 9% oral +insulin.<br><br>Baseline means: HbA1c: 7% (53mmol/mol) BMI: 33 kg/m2, BP: 133/84 mmHg.	Trained mental HCPs (did not contact pt's HCP)	type 2 diabetes education, how to talk with HCPs, diet, exercise, pedometers/FC equipment). Education adapted to population.<br><br>Self-monitoring, modelling practice, goal setting, reinforcement for attending /behaviour change<br>Group-based, face-to-face,	24w education programme lasting 90m/week.<br><br>FU: 6m	Usual care + information from ADA brochures	**Narrative:** No substantial effect on HbA1c at 6 or 12m (12m HbA1c 7.9% (63mmol/mol) (I) Vs 6.9% (52mmol/mol|) (c). No substantial improvement in self-care behaviour or BP. Small improvements in BMI immediately after intervention and at 6m. Self-efficacy improved immediately after intervention.	Small BMI/self-efficacy improvement. Insufficient evidence whether SM for people with severe mental illness improve type 2 diabetes management.<br><br>Very low quality of evidence. Small study number. Results showed inconsistency.<br><br>*Too few RCTs to assess publication bias*
Culturally specific Reviews								
**Attridge 2014 [24]**<br>33 RCTs<br>n=7453<br>Search dates: 2007-2013<br>R-AMSTAR 42	Education that is culturally, religiously and linguistically appropriate to the type 2 diabetes community	Ethnic minorities in deprived areas of upper-middle/high income countries: Hispanic American (14 RCTs), African American (12 RCTs), UK S. Asians (4 RCTs),	Primary-care, hospital clinics, church, home visits 2.5d retreat.<br><br>Delivery: HCPs, CHWs, nurses, dieticians, exercise physiologists, psychologists,	Weight reduction, physical activity, social support, diabetes knowledge, SM and behavioural skills<br><br>Group and individual counselling incorporating purely interactive patient-centred sessions	Median duration: 6m (range: 1-24m)<br><br>FU: Immediately post intervention to 24m. Mode 3-9m	Usual/ conventional education that did not take cultural background into account, e.g. some control groups	**Meta-analysis:** HbA1c at 3m MD-0.4% (95% CI -0.5, -0.2) (-4mmol/mol), 6m MD -0.5% (-5mmol/mol) (95% CI -0.7, -0.4) 12m MD –0.2%, (95% CI -0.3 to -0.04) (-2mmol/mol), 24m MD -0.3% (95% CI -0.6 to -0.1) (-3mmol/mol)	Culturally appropriate health education has positive effects on glycaemic control in the short and medium term and triglycerides in the short term. There is no long-term data to make conclusions about whether these effects are sustained longer term. Results of this update has strengthened

		S. Asians in the Netherlands (1 RCT), Portuguese Canadians (1 RCT), American Samoans (1RCT), Native Americans (1 RCT), US Koreans (1 RCT)	multimedia based interventions e.g. bilingual computer-based learning and social networking	and/or semi-structured didactic formats. Dietary booklets, cassettes, phone counselling, locally developed healthy living programme		received group sessions or nurse home visits.	Triglyceride reduced in the short term only (24 mg/dL (95% CI -40, -8)) Neutral effects on total, HDL, LDL cholesterol, BP and BMI.<br><br>Neutral effect on self-efficacy, QoL, empowerment.	the findings of the original systematic review.<br><br>The heterogeneity of the studies made subgroup comparisons difficult<br><br>*Possible publication bias identified*
**Ferguson 2015 [45]**<br>13 RCTs<br>n=2784<br>Search dates to August 2014<br>R AMSTAR 35	Self-management education in conjunction with primary care among Hispanic adults	93.5% Hispanic. Mean age 47.9 to 70.3y. Majority females in all but 1 study.<br><br>Diabetes duration < 6m to >16y. Baseline HbA1c 7.4% - 11.8% (57mmol/mol-105mmol/mol)	Primary care setting<br><br>Delivery: nurses, peer/diabetes educators, trained clinic employees, multiple providers. (Educators with same cultural background (7 RCTs))	Culturally relevant lifestyle advice mindful of beliefs. Needs of Hispanic community assessed prior to study, type 2 diabetes experience of local leaders.<br><br>Individual, group, phone, video conference sessions, educational videos + FU phone calls, and multi-modal sessions.	Duration: 6w-5y. Total SM provider-patient contact time was 32.6-52h.<br><br>FU 6m-5y	Usual care or "enhanced primary care": access to diabetes educational brochures and phone calls	**Meta-analysis**: pooled HbA1C −0.25 (95% CI, −0.42 to −0.07) (-3mmol/mol) at 6-12m FU (favouring intervention).<br><br>HbA1c affected by cultural tailoring, multimodal strategy design. No significant differences for duration/contact time.	SM education in conjunction with PC modestly improved 6-12m glycaemic outcomes in Hispanic adults.<br><br>Interventions most beneficial when culturally tailored, delivered in range of options by multiple educators working together. Internet and phone interventions alone unsuccessful, but useful in a multimodal approachs.<br><br>*No evidence of publication bias.*
**Choi 2016 [40]**<br>53 RCTs<br>n=8973<br>Search dates: 2004- 2014<br>R-AMSTAR 29	Educational approaches for glycaemic improvements in Chinese diabetes patients.	Chinese patients based in China, Hong Kong and Taiwan (ethnic majority).	NR	Ongoing regular education (didactic lectures). Goal setting/MI (3 RCTs), family education (2 RCTs), phone/SMS coaching (6 RCTs), facilitated peer-learning (6 RCTs), education co-ordinated across settings (6 RCTs), SM written material (4 RCTs), meal/nutrition planning (2 RCTs) diet calculations (3 RCTs)	Intensive short-duration education (3-8w) (8 RCTs), short education (e.g. 30–150m during study period (28 RCTs)<br><br>Median FU: 6m (range 3-18m)	Usual care, no education, self-education,	**Meta-analysis** Overall WMD in HbA1c was 1.19%. (4 RCTs) (13mmol/mol)	Education in any format generates glycaemic improvement for Chinese pts. Suggest recommendations based on Western research may not suit Chinese educational needs. Didactic lectures may be more effective.<br><br>Only 4/111 RCTs used in the HbA1c meta-analysis. Detailed summary tables not provided.<br><br>*No evidence of publication bias.*

**Khunti 2008 [17]**<br>5 RCTs<br>n=1004<br>Search dates: 1997-2006<br>R-AMSTAR 30	Any educational intervention for migrant S. Asian populations	S. Asian populations living in Western countries mainly mixed. 2 RCTs looked only at 1 population: Surinamese and Pakistani.	Delivered by link workers	Tailored clinic sessions + education, enhanced care +education, structured education, flashcards, culture specific care<br><br>one-to-one (4 RCTs), group (1 RCT)	Median FU (included intervention period) 12m	NR	**Narrative** Suggestion of short term improved glycaemic control, less evidence of long term benefit<br><br>Some suggestion of improved BP. Mixed findings for cholesterol, BMI/weight. No difference between group and one to one	Educational interventions for migrant S. Asian populations improved glycaemic control short term, but not long term. Some suggestions of improved BP.<br><br>*Publication bias not assessed*
**Little 2014 [25]**<br>12 RCTs<br>n=2677<br>Search dates to Feb 2014<br>R-AMSTAR 27	CHW delivered interventions for Latino population with type 2 diabetes	Most low-income, Spanish-speaking women with low educational attainment (described as immigrants in 4RCTs).<br><br>Average baselines: HbA1c 7.3 - 10.5%. BMI 30.1-34.4 kg/m2.	CHCs, home visits, CHC +home/phone.<br><br>CHW led education, advocacy (referral to medics, pt–Dr communication), goal-setting, 38pt booking, curriculum development.	SM, knowledge, diet, PhA, medication adherence, advocacy, self-efficacy, foot/eye/dental care, sick day rules, behaviour modification.<br><br>Spanish/literacy tailoring, including family/friends, ethnic foods, prayer, video novella, phone-based vignettes, pedometers	Duration range: 1.5-24m. 6-36 sessions. (Most weekly) lasting 1-2.5h.<br><br>10 RCTs "high intensity": tailored,1-to-1, face-to-face, ≥1h/session, ≥3m length, ≥3 contacts<br><br>FU range: 6-24m	Usual care (+ diabetes management /mental health/diabetes cookbook and quarterly phone calls in one RCT plus bilingual newsletter in another.)	**Narrative:** 7/12 "high intensity" RCTs reported HbA1c improvement (effect sizes from −0.37 to −0.75) at ≥1 FU points ($p<0.05$). 5 of these found no difference at 12m. HbA1c improved (effect sizes at 24m of −0.6, −0.69 ($p<0.05$)) in 3 RCTs with longest FU.<br><br>Behavioural improvements: diet (2 RCTs), PhA (2RCTs), self-efficacy (3 RCTs). No change: lipids, BP, weight (most RCTs)	Mixed evidence on glycaemic control. CHWs held some promise in promoting type 2 diabetes-related behaviour, knowledge and self- efficacy.<br><br>No conclusion for optimal duration/FU<br><br>Good methodological quality but reporting inconsistencies.<br><br>*Potential publication bias identified.*
**Nam 2012 [19]**<br>12 RCTs<br>n=1495<br>Search dates: 1997-2009<br>R-AMSTAR 35	Culturally-tailored diabetes education interventions.	Mean age 63.6y. 68% female. African-American (4RCTs) included, Hispanic Americans (3RCTs), Asians (4RCTs), others (1RCT) Mean baseline HbA1c level: 8.6% (SD 1.4%; median 8.5%)	58% Hospital OPD/ education centre 42% community<br><br>Delivery: 36% nurse 36% dietician. Bilingual/bicultural educator/non-HCP provided education	Culturally appropriate: diet, knowledge, PhA, psychosocial strategy. Preferred language used, low-literacy visual aids, family present in 8 RCTs.<br><br>84% Group ± individual 16% individual only.	Median duration: 3m (One-off - 12m). Contact 1 to >30h.	50% usual care 50% minimal intervention	**Meta-analysis** Overall HbA1c reduction (I Vs C). (ES -0.29). No evidence of long term benefit.	Culturally-tailored interventions improve glycaemic control short term. Community-based interventions may have larger benefits than hospital or clinic based.<br><br>*Potential publication bias identified.*

**Pérez-Escamilla 2008 [55]**<br>2 RCTs<br>n=214<br>Search dates: 1997-2007<br>R-AMSTAR 25	Peer nutrition education and counselling to Latinos delivered by the community.	Puerto Rican and Mexican origin living in USA.	Delivery: bilingual/ bicultural Puerto Rican CHWs living in target community or bilingual clinic employees with 60h type 2 diabetes SM training	Classes and FU calls following ADA guidelines, CHWs liaising with HCPs, (reinforcing self-care and nutrition education).	8 weekly 2h groups classes for 6m. Frequent FU phone contact.	Education without CHW support/ usual care	**Narrative:** Inconclusive mixed effects<br>Improvement in glycaemic control in the 2 RCTs considered. Lipids, bp, knowledge, SM and social support not reported<br>CHWs associated with greater completion rates<br><br>Peer nutrition education has a positive influence on diabetes self-management, breastfeeding outcomes, as well as on general nutrition knowledge and dietary intake behaviours among Latinos	Peer nutrition education had inconclusive mixed effects.<br><br>*Publication bias not assessed*
**Ricci-Cabello 2014 [29]**<br>20 RCTs<br>n=4348 overall<br>n=3280 meta-analysis RCTs<br>n=1068 in non–meta-analysis RCTs<br>Search dates to Oct 2012<br>R-AMSTAR 33	SM educational interventions targeted to racial/ethnic minority groups.	Ethnic minorities (15 RCTs) or low income/ literacy or elderly populations (5 RCTs) in Netherlands, UK, US.<br><br>Meta-analysis: African-American-5 RCTs, Mexican-American-4 RCTs, Multiple ethnicity -3 RCTs, Hispanic -3 RCTs, British–Pakistani- 1 RCT, S Asian -1 RCT, urban -1 RCT.	Meta-analysis: GP -7 RCTs, CC-7 RCTs, home -2 RCTs, clinic -1RCT, hospital -1 RCT<br><br>bilingual MDT- 10 RCTs, community link educators -3 RCTs, solo peer educator -2 RCTs. Dietician-1 RCT, PA coaches – 1 RCT.	Meta-analysis: diet -15 RCTs, PhA -13 RCTs, drug adherence -7 RCTs, basic knowledge -5 RCTs<br><br>Didactic learning -14 RCTs psychological strategy -10 RCTs, situational problem solving -9 RCTs, goal-setting -11 RCTs, cognitive reframing -4 RCTs<br><br>Face to face -11 RCTs, telecommunication -3RCTs, both -4RCTs. Group -6 RCTs, individual-6 RCTs, both -6 RCTs, family invited -5 RCTs.	Median duration: 6m (range 2-24m). Median sessions: 8.5 (range 4 to 52). Median FU was 0m, (range 0-12m).	Usual care	**Meta-analysis** HbA1c decreased by 0.31% (95% CI −0.48%, −0.14%) (3mmol/mol) Meta-regression showed larger reduction in individual, face to face delivery than tele-communication. Peer educators, cognitive reframing were beneficial.<br><br>Most programs obtained some benefits over standard care in improving, knowledge, SM behaviour and clinical outcomes. SM measures too heterogeneous to pool.	Diabetes SM educational programs targeted to racial/ethnic minority groups can produce a positive effect on diabetes knowledge and SM behaviour, ultimately improving glycaemic control.<br><br>*No evidence of publication bias*
**Systematic Reviews facilitated by technology**								

**Arambepola 2016 [23]**<br>13 RCTs<br>n=1155<br>Search dates: to April 2015<br>R-AMSTAR 38	Brief messages via mobile devices promoting healthy eating and increasing PhA in improving glycaemic control.	NR	Remote settings.<br>Messages: unidirectional (from provider/ researcher) or bidirectional (real time automated tailored feedback)	Diet and PhA.<br><br>Providing information, performance feedback, behaviour self-monitoring, rewards, prompts, time/ stress management, goal setting, consequences	Duration/FU: NR<br>Intensity varied from 7 sessions/d to 3 sessions/w. Many dependent on pt preference	Usual care or intervention unlikely to cause any effect	HbA1c decreased by 0.53% (95% CI -0.59% to -0.47%) (6mmol/mol) between intervention and control.<br>BMI NS (5 RCTS)<br>Unidirectional and bidirectional messages produced similar effects.	Automated brief messages strategies can improve health outcomes in people with type 2 diabetes. Trials were not free of bias and did not use explicit theory.<br><br>*No evidence of publication bias*
**Hadjiconstantinou 2016 [39]**<br>10 RCTs<br>n=3612 (includes T1D and type 2 diabetes)<br>Search dates: 1995-2016<br>R-AMSTAR 33	Evaluation of web-based programs/ interventions for emotional management and impact on well-being in type 2 diabetes.	Demographic summary combined T1D and type 2 diabetes so not reported here.<br><br>Based in US and Canada	Delivery: range of HCPs and non-professional providers such as lay people and graduates	Information provision, self-monitoring, feedback for motivation, goal setting, problem solving, action planning, social support, review of goals<br><br>Asynchronous/synchronous communication -6 RCTs, provider/user communication -4RCTs, peer support -3 RCTs	Modal duration:12m<br><br>6-8 sessions, lasting 45-120min	Varied from usual care, enhanced usual care	**Meta-analysis:** Most common behaviour change technique: "general information" and "tracking/monitoring." No significant improvements in depression or distress found by meta-analysis.	Meta-analyses demonstrated non-significant results for depression and distress scores. Potential for Web-based intervention to improve well-being outcomes in type 2 diabetes. Further research is required to confirm the findings of this review.<br><br>*No evidence of publication bias*
**Hou 2016 [37]**<br>10 RCTs<br>n-851<br>Search dates: 1996-2015<br>R-AMSTAR 37	Effect of mobile phone apps on HbA1c in self-management of diabetes.	Mean age 51-62y, mean type 2 diabetes duration: 5-13y, Ethnicity: White -4 RCTs, African American -1 RCT, Afro-Caribbean -1RCT, "Black"-1 RCT, Indo-Asian -1RCT other -2RCTs, NR -6RCTs<br><br>RCTs: Europe -4RCTs, USA -3RCTs, Asia -2 RCTs, Africa -1RCT	PC -3RCTs, CHC -2RCTs, hospital -2RCTs, CHC +hospital -1RCT, community diabetes +PC -1RCT, NR -1RCT	Personalised feedback on data (BP, Wt, BG, diet, PhA-pedometer), HCP feedback when abnormal data -3 RCTs or regular weekly-3-monthly intervals -4 RCTs.	Median duration 4.5m (range 2-12m)<br><br>FU: <6m -5RCTs, >6m -5RCTs	Usual Care, enhanced usual care (+supportive lifestyle intervention)	**Meta-analysis:** Mean reduction in HbA1c in app users Vs control: 0.49% (95% CI 0.30, 0.68) 5mmol/mol with moderate GRADE of evidence.<br>Younger pts more likely to benefit, effect size enhanced with HCP feedback.	Apps may be an effective component to control HbA1c and could be considered as an adjuvant intervention to standard SM. Given clinical effect, access and nominal cost it is likely to be effective at population level.<br><br>*Potential publication bias identified*

**Pal 2013 [27]**<br>16 RCTs<br>n=3578<br>Search dates to Nov 2011<br>R-AMSTAR 41	Computer-based diabetes self-management interventions	Ethnicities included: American Indians, Latino /Hispanic, native Alaskans, White. Mean diabetes duration: 6-13y. Mean age 46-67y.<br><br>3 RCTs involved both T1D (20%) and type 2 diabetes. Authors extracted type 2 diabetes and discarded T1D data where possible.	Clinic -6 RCTs, internet -5 RCTs, online moderated forums (peer support/education) - 4 RCTs. Mobile devices – 5 RCTs	Computers assessed diet/ PhA barriers, provided SM education/tailored diet action plans, online peer support (moderated forum). Mobile reminder: medication, SMBG, weight /BP measurement, meal-time/PhA reinforcement, lab results, custom message + response function, tailored lifestyle advice texts from HCPs.	Median duration: 5.5m (range:1-12m)<br>Low intensity: 1-4 doses -6 RCTs, >2 interactions/d –3 RCTs, participant-driven exposure, frequency, intensity -7 RCTs.<br><br>FU range 2-12m, <1m -0, 1-6m -11 RCTs, >6m -5 RCTs	Usual care, Non-interactive computer programme, paper resources, delayed start/ waiting list.<br>Face-to-face education.	**Meta-analysis:** Pooled HbA1c effect: -0.2% (95% CI -0.4, -0.1) (p = 0.009). (2mmol/mol)<br>Larger effect size in mobile phone group: MD HbA1c -0.5%, (95% CI -0.7 to -0.3) -5mmol/mol p < 0.00001; 280 pts; 3 RCTs<br><br>Inadequate evidence for improving depression, HRQoL or weight. 4/10 RCTs showed beneficial effects on lipids.	Computer-based diabetes SM interventions have a small beneficial effect on blood glucose control with larger effect in mobile phone subgroup. No evidence to show benefits in other biological outcomes e.g. weight loss or any cognitive, behavioural or emotional outcomes, but they do appear to be safe.<br><br>*Too few studies for meaningful assessment of publication bias*
**Saffari 2014 [30]**<br>10 RCTs<br>n= 960<br>Search dates: 2003- 2013<br>R-AMSTAR 32	Delivery of diabetes health education by mobile phone SMS	Average age: 52.8y, majority women. Average diabetes duration: 7.3y.<br><br>80% RCTs in Asia; Bahrain, India, Iran, Korea, Taiwan, rest USA	Hospital -6 RCTs, CHC -1 RCT, mixed -1 RCT, diabetes association -1 RCT, diabetes clinic -1 RCT.	Interactive SMS sent and received -6 RCTs, SMS received only -4 RCTs, website +SMS for sending /receiving data -4 RCTs	Median duration: 3m (range 3-12m)<br><br>FU: 3m -6 RCTs	NR	**Meta-analysis:** Significant HbA1c reduction compared to control. (SMD -0.6 (95% CI -0.83, -0.36) -6 mmol/mol p<0.001).<br>Effect size in SMS-only group was 44%, this increased to 86% (p=0.002) in studies using SMS and internet.<br>SMD more statistically significant when HbA1c <8%.	Educational mobile SMS improved glycaemic control. Multimedia approach may increase effect. Interactive data gathering/provision may reduce HbA1c more than uni-directional. Pts <55y had greater HbA1c declines.<br>*Possible publication bias*
**Tao 2013 [33]**<br>24 RCTs<br>n=6489 (includes T1D)<br>Search dates to July 2012<br>R-AMSTAR 29	Evaluation of self-management health information technology (SMHIT) on glycaemic control	Not extracted as T1D and type 2 diabetes not differentiated	Home, no location restrictions, clinics, CHCs, medical centres. NB: T1Dm and type 2 diabetes not differentiated	Computer and or mobile phone –based SMHIT<br><br>No further extraction as T1D and type 2 diabetes not differentiated.	≤3m -12 RCTs, 4-11m -18 RCTs, ≥12m -13 RCTs. NB: T1D and type 2 diabetes not differentiated for duration	NR	**Meta-analysis**: SMHIT-assisted intervention group had larger reductions in HbA1c than control (SMD -0.36%, -4mmol/mol p <0.001).	Web based interventions show favourable outcomes for type 2 diabetes<br><br>*Possible publication bias*

**Van Vugt 2013 [34]**<br>7 RCTs<br>n=2400<br>Search dates: 1994-2012<br>R-AMSTAR 25	Application of BCT in online SM programs for type 2 diabetes.	Mean age 57.6y (range 54.3-59.3y) mean 54% female (range 53-73%)<br>Ethnicity: White -4 RCTs Asian -1 RCT, Native Alaskan -1 RCT, Native Indian -1 RCT, White-Latino -1 RCT	PC -5 RCTs, secondary care -1 RCT, mixed -1 RCT<br><br>HCP involved -4 RCTs, no HCP involved -3 RCTs	All RCTs web-based. +2 FU calls +3 group sessions -2 RCTs, +online forum -3RCTs.<br><br>Goal setting/action plan -6 RCTs, feedback -6 RCTs, MI +tailored PhA advice -1 RCT, community resources -2 RCTs.	Duration mean 7.5m (range 3-18m)<br><br>FU: NR	usual care, enhanced usual care, online diabetes information	**Narrative**: statistically significant improvements in: HbA1c, fasting blood glucose, cholesterol, and triglycerides -6/7 RCTs. Health behaviour (diet, PhA medication use, smoking) -5/7 RCTs, psychological outcomes e.g. depression, distress, self-efficacy -5 RCTs. Goal setting linked to improved clinical outcome, facilitating social comparison linked to improved psychological outcomes.	Potentially effective BCTs rarely used in online self-management programs despite a good theoretical basis. Only a few social theory BCTs, which have a great influence on the self-management of type 2 diabetes, were represented in the studies claiming to use them.<br><br>*Publication bias not assessed*

Abbreviations: RCTs: Randomised Control Trials, WM: Weighted Mean, Y: years, SysBP: systolic blood pressure. BMI: body mass index, DM: Diabetes mellitus, PAI: Patient Activation Intervention, M: months, LDL: low density lipoprotein, CI: confidence Interval , LTC: long term condition, SM: self-management, NR: not reported, UK: United Kingdom, USA: United States of America, R-AMSTAR: Revised Assessment of Multiple Systematic Reviews, ES: effect size, CHW: Community heath-care worker, SW: Social Work , FU: Follow- up, W: weeks, I: intervention, C: control, d: days, BP: blood pressure, MI: motivational interviewing, PA: physician assistant, OPD: out-patient department, AV: audiovisual, SMBG: Self-monitoring blood glucose, Wt: weight, HRQoL: health related quality of life, SMD: Standardized mean difference, GHb: glycated haemoglobin, HCW: Health-care worker, PhA: physical Activity, ADA: American Diabetes Association, pt: patient, PC: primary care, NS: not significant, ED (emergency department), FC: footcare, DKD: diabetes kidney disease, PD: Peritoneal dialysis, HD: haemodialysis, apt: appointment, CHC: community health centre CC: community centre, SMS: short message service, SMHIT: self-management health information technology, BCT: behaviour change technique

Table 5: Quality assessment using R-AMSTAR scoring

	1. Was an a priori design provided?	2. Was there duplicate study selection and data extraction?	3. Was a comprehensive literature search performed?	4. Was the status of publication used as an inclusion criterion?	5. Was a list of studies (included and excluded) provided?	6. Were the characteristics of the included studies provided?	7. Was the scientific quality of the included studies assessed and documented?	8. Was the scientific quality of the included studies used appropriately in formulating conclusions?	9. Were the methods used to combine the findings of studies appropriate?	10. Was the likelihood of publication bias assessed?	11. Was the conflict of interest included?	Total score /44	Population size
**Fan 2009**	3	1	4	1	1	3	1	1	1	1	3	20	
**Newman 2004**	4	1	3	2	1	3	2	4	1	1	1	23	2032
**Heinrich 2010**	4	1	4	2	2	3	2	1	1	1	3	24	1778
**Pérez-Escamilla 2008**	4	1	3	1	4	3	3	2	1	1	2	25	214
**Sigurdardottir 2007**	4	1	4	1	2	2	4	4	1	1	2	26	4293
**Van Vugt 2013**	4	4	3	1	2	4	4	1	1	1	2	27	2400

**Norris 2001**	4	1	4	1	1	2	4	3	4	1	2	27	NR
**Little 2014**	4	3	4	1	2	4	2	2	1	2	2	27	2677
**Song 2014**	4	4	4	2	1	2	3	1	4	1	2	28	2947
**Choi 2016**	3	4	3	2	2	2	2	1	4	3	3	29	8973
**Khunti 2008**	4	4	4	4	3	3	4	1	4	1	3	30	1004
**Norris 2002**	4	1	4	2	3	2	3	4	4	2	2	31	4263
**Van Dam 2005**	4	1	4	3	1	3	4	4	4	1	2	31	712
**Chrvala 2016**	4	4	4	2	2	4	4	2	2	1	2	4	22947
**Ekong 2016**	4	4	4	1	2	3	3	2	4	1	3	4	4066
**Jonkman 2016**	4	3	4	2	2	4	2	1	4	3	2	31	3829
**Dale 2012**	4	4	4	2	1	3	4	2	4	1	3	32	3763
**Saffari 2014**	4	3	3	1	2	4	3	2	4	4	2	32	960
**Tao 2013**	4	4	3	1	2	4	3	2	4	4	2	33	6489*
**Ricci-Cabello 2014**	4	4	4	3	2	3	2	1	4	3	3	33	3094
**Sherifali 2016**	4	4	4	1	2	4	4	2	4	1	3	33	724
**Hadjiconstantinou 2016**	4	4	4	1	2	4	3	1	4	4	2	33	3612
**Zhang 2016**	4	2	4	1	2	4	3	3	4	4	2	33	4494

**Chodosh 2005**												34	2579
**Patil 2016**	4	4	4	2	2	3	3	1	4	4	3	34	4715
**Nam 2012**	4	1	4	2	2	4	4	3	4	4	3	35	1495
**Ferguson 2015**	4	3	4	1	2	4	4	2	4	4	3	35	2784
**Qi 2015**	4	4	3	1	2	4	3	3	4	4	3	35	2352
**Duke 2009**												36	1359
**Gary 2003**	4	2	3	1	2	4	4	2	3	3	2	36	2720
**Pillay 2015**	4	3	4	2	4	4	4	1	4	3	4	37	8715
**Hou 2016**	4	4	4	2	2	4	4	4	4	3	2	37	851
**Minet 2010**												37	7677
**Steinsbekk 2012**	4	4	4	3	3	4	4	3	4	1	3	37	2833
**Arambepola 2016**	4	4	4	2	3	4	3	2	4	4	4	38	1155
**Bolen 2014**	4	4	4	1	3	4	4	4	4	4	3	39	33124
**Dorresteijn 2012**	4	4	4	4	4	3	4	2	4	3	3	39	3167
**Li 2011**												41	207
**McBain 2016**	4	4	3	4	4	4	4	3	4	4	3	41	64
**Pal 2013**	4	4	4	4	4	4	4	3	4	3	3	41	3578

**Attridge 2014**	4	4	4	4	4	4	4	4	4	3	3	42	7543

Table 6 Summary of meta-analysis findings

Reference	Outcome	Follow-up (months)	N RCTs	N participants	Signifi-cance	Summary of results
Arambepola 2016	HbA1c (%) (bidirectional messages)	NR	5	381	+	WMD -0.52 (CI -0.69, -0.34)
	BMI (kg/m2)	NR	5	406	0	MD-0.25 (CI-1.02 to 0.52)
Attridge 2014	Primary Outcomes					
	HbA1c (%)	3	14	1442	+	MD -0.39 (CI-0.64, -0.13)
	HbA1c (%)	6	14	1972	+	MD -0.53 (CI -0.72, -0.35)
	HbA1c (%)	12	Nine	1966	+	MD -0.19 (CI-0.34, -0.04)
	HbA1c (%)	24	Four	2268	+	MD -0.33 (CI -0.61, -0.06)
	HbA1c (%)	Overall	28	5724	+	MD-0.30 (CI -0.38, -0.22)
	HRQoL	3	2	104	0	SMD 0.36 (CI -0.03, 0.75)
	HRQoL (also NS at 12 months)	6	3	224	0	SMD 0.19 (CI -0.08, 0.45)
	Secondary Outcomes:					
	Self-efficacy (also NS at 12 months)	3	6	720	0	SMD 0.06 (CI -0.14, 0.26)
	Self-efficacy	6	4	903	+	SMD 0.49 (CI 0.18, 0.80)
	Mean total chol (also NS at 3, 6 months)	12	5	1019	0	MD-5.84 (CI -13.19, 1.51)
	Mean LDL (also NS at 3, 6 months)	12	3	687	0	MD -0.13 (CI -5.72, 5.45)
	Mean HDL (also NS at 3, 6 months)	12	3	471	0	MD 0.32 (CI -1.67, 2.31)
	Mean triglycerides	3	5	662	+	MD -23.98 (CI -39.73, -8.23)
	Mean triglycerides (also NS at 6 months)	12	3	584	0	MD -5.55 (CI -25.53, 14.42)
	BMI (BMI NS at all time points)	12	2	358	0	MD -0.38 (CI -1.70, 0.95)
	Systolic BP	12	5	1209	0	MD 1.43 (CI -0.96, 3.81)
	Diastolic BP	12	4	886	0	MD 0.06 (CI -2.82, 2.93)
Bolen 2014	Primary outcomes					
	HbA1c (%)	3 <24	111	12780	+	WMD -0.37 (CI-0.45 to -0.28)
*long term: >2 years of follow up	HbA1c <8%	3<24	55	NR	+	WMD -0.28 (CI-0.40 to -0.16)
	HbA1c ≥8%	3<24	56	NR	0	WMD -0.48 (CI-0.60 to 0.35)
	Secondary outcomes					
	SBP (mmHg)	3<24	54	7630	+	WMD -2.2 (CI-3.5 to -1.0)
	SBP <137 mmHg)	3<24	26	NR	0	WMD -1.3 (CI-3.0 to 0.4)
	SBP ≥137 mmHg)	3<24	28	NR	+	WMD -2.9 (CI-4.7 to -1.2)
	LDL (mg/dL(	3<24	37	4845	+	WMD -4.2 (CI-6.9 to -1.5)
	LDL <112mg/dL	3<24	18	NR	0	WMD -2.6 (CI-5.4 to 0.1)
	LDL ≥112mg/dL	3<24	19	NR	+	WMD -5.6 (CI-10 to -1.3)
	HDL- Cholesterol (mg/dL)	3<24	34	4908	0	WMD 0.03 (CI-0.8 to 0.8)
	HDL-C <46.5mg/dL	3<24	17	NR	0	WMD -0.2 (CI-1.1 to 0.6)
	HDL- C ≥46.5mg/dL	3<24	17	NR	0	WMD 0.12 (CI-1.2 to 1.5)
	Triglycerides (mg/dL)	3<24	38	5021	+	WMD -8.5 (CI-15.0 to -2.3)
	TG <176 mg/dL	3<24	19	NR	+	WMD -9.2 (CI-18.3 to -0.1)
	TG≥176 mg/dL	3<24	19	NR	-	WMD -4.2 (CI-11.6 to 3.2)
	Body Weight (lbs)	3<24	43	5749	+	WMD -2.3 (CI-3.2 to -1.3)
	BW <202 lbs	3<24	20	NR	+	WMD -2.5 (CI-3.9 to -1.1)
	BW≥202 lbs	3<24	23	NR	+	WMD -2.0 (CI-3.4 to -0.6)
	Mortality	>24	6	2733		OR 0.70 (0.49, 1.01)
	CVD Morbidity	>24	1	141		RD: 20% less in IG
	Nephropathy	>24	1	141		RD: 10% less pts w proteinuria & 4% less ESRD in IG
	Retinopathy	>24	2	251		RD: 20% more control pts developed retinopathy
	Mortality	<24				OR 5.4 (1.2 to 25.1)
	Mortality	3<24	38	8791		OR 0.85 (0.61 to 1.17)
Chodosh 2005	HbA1c	NR	20	NR	+	ES -0.36 (CI -0.52 to -0.21)
	Fasting blood glucose	NR	13	NR	+	ES -0.28 (CI -0.47 to -0.08)
	Weight	NR	17	NR	0	ES -0.04 (CI -0.16 to 0.07)
Choi 2016	HbA1c (intervention Vs control) (%)	5-12	4	544	+	WMD -1.19 (CI -1.92, -0.46)
	HbA1c (intervention group) (%)	NR	68	5565	+	WMD -1.75 (CI-1.96, -1.53)
	HbA1c (control group) (%)	NR	34	3029	+	WMD -0.87 (CI -1.15, -0.6)

Duke 2009	*Comparison 1: Individual education Vs Usual Care*					
	HbA1c (%)	<12	3	295	0	WMD -0.2(CI -0.05, 0.03)
	HbA1c (%)	≥12	4	632	0	WMD -0.1(CI -0.3, 0.1)
	SBP (mmHg)	≥12	3	625	0	WMD -2 (CI -5, 1)
	DBP (mmHg)	≥12	3	624	0	WMD -2 (CI -3, 0)
	Cholesterol (mmol/l)	≥12	3	627	0	WMD -0.03 (CI-0.2, 0.1)
	BMI (kg/m2)	≥12	2	312	0	WMD -0.2 (Cl -1.0, 0.62)
	Comparison 2: Individual education Vs Group education					
	Primary Outcome					
	HbA1c (%)	<12	2	148	+	WMD 0.8 (CI 0.3 to 1.3)
	HbA1c (%)	≥12	2	112	0	WMD 0.03 (CI -0.02, 0.1)
	Secondary Outcomes					
	SBP (mmHg)	≥12	2	95	0	WMD 4.0 (CI -4, 12)
	DBP (mmHg)	≥12	2	95	0	WMD 2.0 (CI -4, 7)
	BMI (kg/m2)	<12	2	169	0	WMD -0.1 (CI -0.9, 0.7)
	BMI (kg/m2)	≥12	2	123	0	WMD-0.01 (CI-0.8, 0.7)
Fan 2009	Fasting blood glucose	NR	15	NR	+	WMES 0.56 (range 0.29-0.89)
	SBP	NR	12	NR	+	WMES 0.57 (range 0.30-0.83)
	DBP	NR	10	NR	+	WMES 0.66 (range 0.27, 1.05)
	Cholesterol	NR	20	NR	+	WMES 0.52 (range 0.26, 0.78)
	Triglycerides	NR	15	NR	+	WMES 0.25 (range 0.10, 0.41)
	BMI	NR	34	NR	+	WMES 0.08 (range 0.12, 0.43)
	Overall self-management behaviours (based on diet, exercise, SMBG, medication, recognition of complications, foot care)	NR	68	NR	+	WMES 0.36 (range 0.30-0.43)
Ferguson 2015	HbA1c (%)	6-12	11	2616	+	SMD-0.25 (CI-0.42, -0.07)
Gary 2003	GHb (total Ghb, HbA1, HbA1c)	NR	18	NR	+	WMD -0.43 (CI-0.71, -0.14)
	Fasting blood glucose (mg/dl)	NR	12	NR	0	WMD -12.22 (CI-25.1, 0.67)
	Total GHb (%)	NR	6	NR	0	WMD -0.4 (CI-0.73, 0.08)
	HbA1 (%)	NR	7	NR	0	WMD -0.77 (CI-1.88, 0.34)
	HbA1c (%)	NR	5	NR	+	WMD -0.52 (CI-0.96, -0.08)
	Weight (lbs)	NR	7	NR	0	WMD -4.64 (CI -9.95, 0.66)
Hadjiconstantinou 2016	Depression score	NR	5	NR	0	MD -0.31 (CI -0.73 to 0.11)
	Distress score	NR	6	NR	0	MD -0.11 (CI -0.38, 0.16)
Hou 2016		Overall	10 RCTs	851	+	MD -0.49 (-0.3, - 0.68)
		<6	5 RCTs	NR	0	MD -0.62 (NR)
		>6	5 RCTs	NR		MD -0.40 (NR)
Jonkman 2015	HRQoL	2-8	11	NR	NR	SMD 0.11 (CI 0.01, 0.22)
	HRQoL	12-24	8	NR		SMD 0.08 (CI 0.02, 0.18)
Minet 2010	HbA1c (%)		47	7677	+	MD 0.36 (0.21, 0.51)
Nam 2012	HbA1c (%)	Overall	12	NR	+	ES -0.29 (CI-0.46, -0.13)
	HbA1c (%)	3	8	NR	0	ES -0.21 (CI-0.47, 0.05)
	HbA1c (%)	6	5	NR	+	ES -0.41 (CI-0.61, -0.21)
	HbA1c (%)	≥12	2	NR	0	ES -0.14 (CI-0.39, 0.11)
Norris 2002	GHb (%) Interventions Vs control group	Immediate	20	2094	+	Net change-0.76 (CI -0.34, 1.18)
	GHb (%)Interventions Vs control group	1-3	9	NR	0	Net change-0.26 (CI 0.21, -0.73)
	GHb (%)Interventions Vs control group	≥4	8	NR	+	Net change-0.26%(CI -0.05, -0.48)
Pal 2013	Primary Outcomes					
	HbA1c (%) total pooled effect	NR	11	2637	+	MD-0.21 (CI-0.37,-0.05)
	HbA1c %	<6	5	842	+	MD-0.32 [CI-0.58, -0.07]
	Hba1c %	>6	6	1795	0	MD -0.14 [CI-0.33, 0.05]
	Weight	NR	3	253	0	SMD-0.05(CI-0.22, 0.13)
	BMI	NR	1	130	0	SMD-0.06 (CI-0.31, 0.19)
	Total cholesterol	NR	4	567	0	MD-0.19 [CI-0.41, 0.02]
	HDL	NR	2	446	0	MD-0.01 [CI-0.08, 0.05]
	LDL	NR	1	NR		Not selected
	TC:HDL ratio	NR	3	1466	0	MD 0.05 [CI-0.07, 0.16]
	Pooled effect on cholesterol	NR	7	1625	0	MD-0.11 [CI-0.28, 0.05]

Patil 2016	HbA1c (%)	NR	17	4715	+	MD 0.24 (CI 0.05, 0.43)
Pillay 2015	HbA1c (%)	0	66 *	8715	+	MD -0.35 [CI -0.56, -0.14]
		6	23	4138	0	MD -0.16 [CI -0.36, 0.04]
		12	9	1494	0	MD -0.14 [CI -0.4, 0.12]
	BMI kg/m^2	0	36	4280	+	MD -0.51 [-0.66, -0.36]
		6	14	1840	+	MD -0.21 [-0.32, -0.01]
		12	5	867	+	MD -0.92 [-1.44, -0.04]
	Total cholesterol (mmol/L)	0	27	2633	+	MD -0.1 [-0.11, -0.09]
		6	7	686	+	MD -0.24 [-0.39, -0.09]
		12	1	291	0	MD -0.10 [-0.34, 0.14]
	HDL (mmol/L)	0	25	2733	+	MD 0.02 [0.02, 0.02]
		6	7	686	+	MD -0.24 [-0.39, -0.09]
		12	1	291	0	MD 0.00 [-0.20, 0.20]
	LDL (mmol/L)	0	27	3063	+	MD-0.03 [-0.03, -0.03]
		6	5	457	0	MD -0.19 [-0.47, 0.09]
		12	1	291	0	MD -0.00 [-0.09, 0.09]
	Triglycerides (mmol/L)	0	24	2561	+	MD -0.17 [-0.24, -0.10]
		6	5	712	0	MD -0.18 [-0.37, 0.01]
		12	1	291	0	MD -0.20 [-0.45, 0.05]
	SBP (mmHg)	0	36	4776	+	MD -0.78 [-1.30, -0.26]
		6	10	1613	0	MD -1.08 [-2.90, 0.74]
		12	1	291	0	MD -2.80 [-7.69, 2.09]
	DBP (mmHg)	0	33	4583	+	MD -0.94 [-1.32, -0.56]
		6	7	1424	+	MD -1.26 [-1.97, -0.55]
		12	1	291	0	MD -2.20 [-4.73, 0.33]
	QoL SF-36 (physical) (higher score desirable)	0	5	787	0	MD 0.45 [-0.05, 0.95]
	QoL SF-36 (mental) (higher score desirable)	0	5	787	0	MD 1.60 [-1.96, 5.16]
	Diabetes QoL -PAID (lower score desirable)	0	8	1384	+	MD -1.82 [-3.43. -0.21]
		6	4	1382	0	MD -1.89 [-4.37. 0.59]
		12	3	757	0	MD -1.30 [-5.84, 3.24]
	Mortality	0	25	4659	0	RR 1.28 [0.84, 1.94]
Qi 2015	HbA1c (%)	NR (60% RCTs >3)	13	2352	+	MD−0.57 (CI −0.78. −0.36)
Ricci-Cabello 2014	HbA1c (%)	Overall	20	3280	+	MD -0.31 (CI -0.48, - 0.14)
	HbA1c (%)	6	3		0	MD−0.47 (CI NR)
Saffari 2014	HbA1c (%)	NR	10	960	+	SMD-0.60 (CI-0.83, -0.36)
Sherifali 2016	HbA1c (%)	3-16	8	724	+	MD-0.32 (CI−0.50, −0.15)
Song 2014	HbA1c (%)	0-6	10	2947	+	WMD -0.29(CI-0.47, -0.11)
	Self-management effects	0-6	3	2346	+	WMD 2.37 (CI 1.77, 2.98)
Steinsbekk 2012	HbA1c (%)	<12	13	1883	+	MD -0.44 (CI -0.69, -0.19)
	HbA1c (%)	12	11	1503	+	MD -0.46 (CI -0.74, -0.18)
	HbA1c (%)	24	3	397	+	MD -0.87 (CI-1.25, -0.49)
	Fasting blood glucose (mmol/l)	<12	3	401	0	NR
	Fasting blood glucose (mmol/l)	≥12	5	NR	+	MD -1.26 (CI-1.69, -0.83)
	QoL	<12	3	473	0	SMD 0.31 (CI-0.15, 0.78)
	Secondary Outcomes					
	Self-efficacy	<12	2	326	+	SMD -0.28 (CI 0.06, 0.5)
	Self-management behaviours	<12	4	534	+	SMD 0.55 (CI 0.11, 0.99)
	SBP (mmHg)	<12	5	815	0	MD -0.34 (CI -5.19, 4.51)
	SBP (mmHg)	≥12	2	NR	0	MD-3.0 (CI-7,2)
	DBP (mmHg)	<12	5	815	0	MD-0.46 (CI-2.31, 1.39)
	DBP (mmHg)	≥12	2	NR	0	MD 0.17 (CI-4.46, 4.80)
	Total cholesterol (mmol/l)	<12	7	1161	0	MD -0.06 (CI-0.23, 0.12)
	Total cholesterol (mmol/l)	≥12	4	NR	0	MD 0.07 (CI -0.09, 0.2)
	Triglycerides (mmol/l)	<12	7	1161	0	MD -0.05 (CI-0.19, 0.08)
	Triglycerides (mmol/l)	≥12	4	NR	0	MD 0.03 (CI-0.42, 0.48)

	HDL (mmol/l)<br>LDL (mmol/l)<br>Body weight (kg)<br>Body weight (kg)<br>BMI (kg/m2)<br>BMI (kg/m2)<br>Mortality	<12<br><12<br><12<br>≥12<br><12<br>≥12<br>NR	6<br>6<br>3<br>4<br>7<br>7<br>NR	932<br>932<br>433<br>492<br>1159<br>1092<br>NR	0<br>0<br>0<br>+<br>0<br>0<br>0	MD 0.01 (CI-0.05, 0.03)<br>MD 0.05 (CI-0.2, 0.1)<br>MD -2.08 (CI-5.55, 1.39)<br>MD -1.66 (CI-3.07, -0.25)<br>MD -0.21 (CI-0.86, 0.43)<br>MD -0.22 (CI-1.13, 0.69)<br>OR 1.10 (CI 0.37, 3.29)
Tao 2013	HbA1c (%) (Type 2 diabetes sub analysis)	NR	32	NR	+	MD-0.36 (CI-0.48, -0.24)
Zhang 2016	HbA1c<br>HbA1c<br>HbA1c	Overall<br>1-6<br>>6	20<br>5<br>3		+<br>0<br>0	WMD-0.16 (CI-0.25, -0.007<br>WMD -0.06 (CI-0.26, 0.15)<br>WMD 0.01 (CI-0.32, 0.34)

Significant finding denoted by + and non-significant denoted by 0.
*comparisons
Abbreviations: HRQoL: health related Quality of Life, BMI: body mass index, chol: cholesterol, LDL: low density lipoprotein, HDL: high density lipoprotein, BP: blood pressure, RCTs: randomised controlled trial, ppts: participants, NS: non-significant, MD: mean difference, CI: confidence intervals, SDM: standard difference in mean, SMBG: self-monitoring of blood glucose, WMES: weighted mean effect size

Supplemental Figure 1: Overlap of randomised controlled trials within the included systematic reviews

	Arambepola 2016	Attridge 2014	Bolen 2014	Chodosh 2005	Choi 2016	Chrvala 2016	Dale 2012	Dorresteijn 2014	Duke 2009	Ekong 2016	Ferguson 2015	Gary 2003	Hadjiconstantinou 2016	Heinrich 2010	Hou 2016	Jonkman 2016	Khunti 2008	Li 2011	Little 2014	McBain 2016	Minet 2010	Nam 2012	Newman 2004	Norris 2001	Norris 2002	Pal 2013	Perez-Escamilla 2008	Qi 2015	Ricci-Cabello 2014	Saffari 2014	Sherifali 2016	Sigurdardottir 2007	Song 2014	Steinsbekk 2012	Tao 2013	Van Dam 2005	Van Vugt 2013	Zhang 2016
Arambepola 2016	14																																					
Attridge 2014	0	33																																				
Bolen 2014	2	15	138																																			
Chodosh 2005	0	2	3	26																																		
Choi 2016	0	0	1	0	53																																	
Chrvala 2016	0	19	51	3	4	120																																
Dale 2012	0	2	0	1	0	6	10																															
Dorresteijn 2014	0	0	2	0	0	0	0	12																														
Duke 2009	0	1	3	1	1	5	0	0	9																													
Ekong 2016	0	1	4	0	1	2	0	0	0	14																												
Ferguson 2015	0	7	8	1	0	10	1	0	0	0	13																											
Gary 2003	0	1	3	4	0	1	0	2	2	0	0	18																										
Hadjiconstantinou 201	0	0	3	0	0	3	1	0	0	0	0	0	10																									
Heinrich 2010	0	2	8	1	0	11	0	0	2	1	0	0	0	14																								
Hou 2016	3	0	2	0	0	0	0	0	0	0	0	0	0	0	10																							
Jonkman 2016	0	1	6	0	0	10	0	0	2	0	1	0	0	1	0	13																						
Khunti 2008	0	3	0	0	0	1	0	0	2	0	0	0	0	0	0	0	5																					
Li 2011	0	0	1	0	0	2	0	0	0	0	0	0	0	1	0	0	0	2																				
Little 2014	0	6	5	0	0	6	1	0	0	0	7	0	0	0	0	0	0	0	12																			
McBain 2016	0	0	0	0	0	0	0	0	0	0	0	0	0	0	0	0	0	0	0	1																		
Minet 2010	0	7	17	1	1	29	1	0	8	1	2	1	0	1	0	3	0	1	0	0	43																	
Nam 2012	0	10	2	1	0	8	0	0	2	0	2	1	0	2	0	2	3	0	0	0	4	12																
Newman 2004	0	2	4	4	0	4	0	0	2	1	1	2	2	0	0	0	0	0	0	0	5	1	21															
Norris 2001	0	2	11	16	0	2	1	10	4	0	0	9	0	0	0	0	1	0	0	0	6	3	3	72														
Norris 2002	0	2	7	8	0	2	0	4	4	0	0	7	0	0	0	0	1	0	0	0	6	3	3	30	31													
Pal 2013	2	0	6	0	0	4	1	0	0	0	0	0	2	1	3	2	0	0	0	0	0	0	0	2	1	16												
Perez-Escamilla 2008	0	0	0	0	0	1	0	0	0	0	1	0	0	0	0	0	0	0	1	0	0	0	0	0	0	0	2											
Qi 2015	0	4	5	0	0	8	4	0	0	0	4	0	0	0	0	0	0	0	4	0	0	0	0	0	0	0	1	13										
Ricci-Cabello 2014	0	9	6	1	0	11	1	0	1	0	4	1	0	1	0	1	1	0	3	0	5	6	1	2	2	0	1	3	20									
Saffari 2014	4	0	1	0	0	0	0	0	0	0	0	0	0	0	2	0	0	0	0	0	0	0	0	0	0	1	0	0	0	10								
Sherifali 2016	1	0	3	0	0	4	0	0	1	0	1	0	0	1	1	1	0	0	0	0	1	0	0	0	0	0	0	0	1	0	8							
Sigurdardottir 2007	0	1	3	2	0	6	0	0	4	0	1	0	0	1	0	0	0	0	0	0	7	2	5	3	3	0	0	0	1	0	0	18						
Song 2014	0	0	1	0	0	0	0	0	0	6	0	0	0	0	0	0	0	0	0	0	1	0	0	0	0	0	0	0	0	0	0	0	10					
Steinsbekk 2012	0	3	7	1	0	9	0	0	0	0	3	0	0	4	0	2	1	0	1	1	7	2	2	3	3	0	1	1	3	0	0	4	0	21				
Tao 2013	2	1	11	1	0	4	1	0	0	0	0	0	3	0	3	2	0	0	0	0	1	0	2	0	0	6	0	0	0	4	0	0	0	0	22			
Van Dam 2005	0	1	1	1	0	2	1	0	0	0	0	0	0	0	0	0	0	0	0	0	2	0	2	2	1	0	0	0	1	0	0	1	0	1	0	6		
Van Vugt 2013	0	0	4	0	0	2	1	0	0	0	0	0	3	0	0	0	0	0	0	0	0	0	1	2	0	3	0	0	0	0	0	0	0	0	3	1	7	
Zhang 2016	0	3	6	0	0	8	6	0	0	0	4	0	0	0	0	0	0	0	5	0	0	0	0	0	0	0	1	9	4	0	1	0	0	1	0	0	0	20

*Shaded systematic reviews correspond to interventions focused on a cultural group

Supplemental Figure 2: Meta-Forest plot of mean difference in HbA1c values according to whether they specified a usual care or a minimal intervention comparator

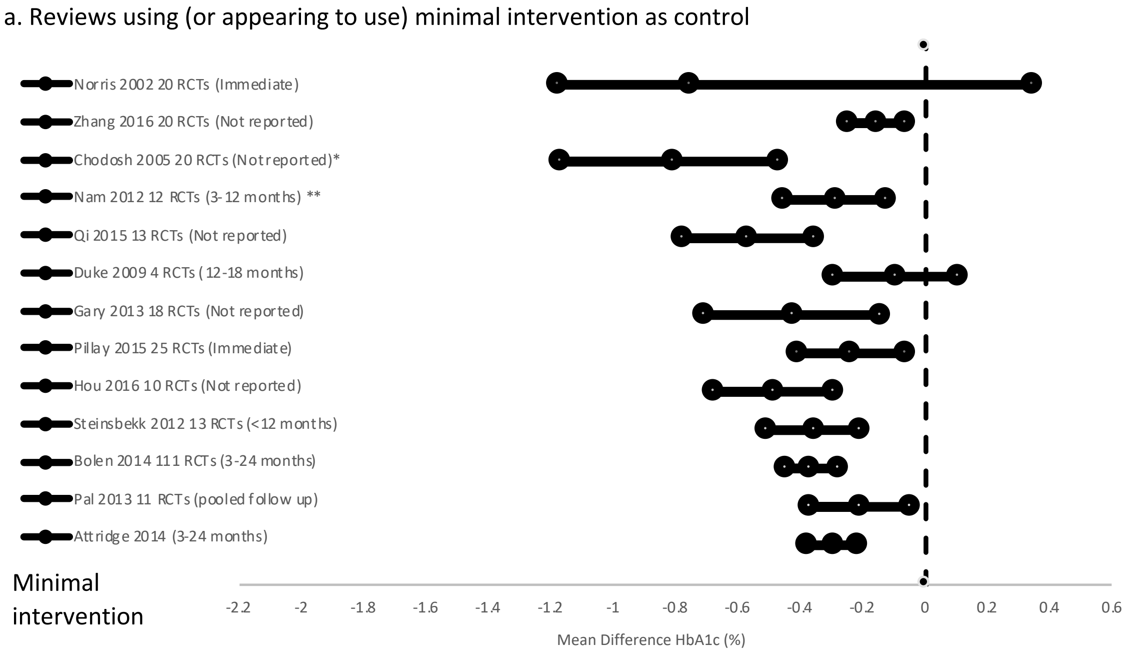

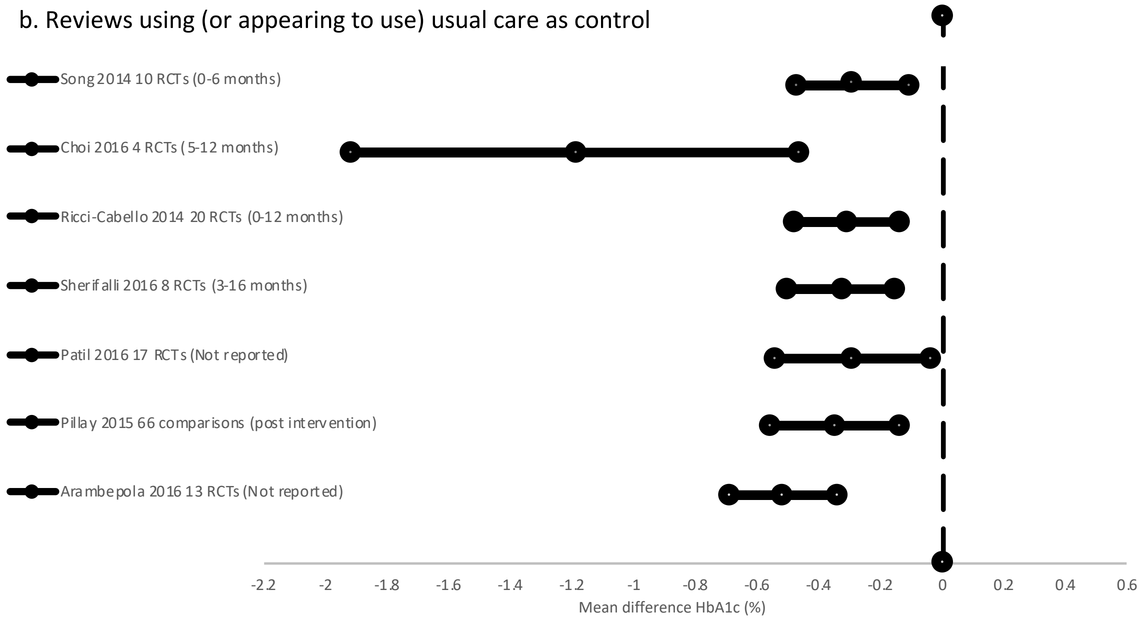

Supplement: Supplementary file 1 [file bmjopen-2018-024262supp001.pdf]
